# Materials Selection, Environmental Ageing, and Circular Design of Canine Vehicle Restraint Systems: A Critical Review

**DOI:** 10.3390/ma19214679

**Published:** 2026-11-03

**Authors:** Damian Frej, Mirosław Gidlewski, Marek Jaśkiewicz, Jerzy Jackowski, Milos Poliak

**Affiliations:** 1 Department of Automotive Engineering and Transport, Kielce University of Technology, 7 Tysiaclecia Panstwa Polskiego Ave., 25-314 Kielce, Poland; mjaskiewicz@tu.kielce.pl; 2 Institute of Vehicles and Transportation, Faculty of Mechanical Engineering, Military University of Technology (WAT), Ave. gen. Sylwestra Kaliskiego 2, 00-908 Warsaw, Poland; miroslaw.gidlewski@wat.edu.pl (M.G.); jerzy.jackowski@wat.edu.pl (J.J.); 3 Department of Road and Urban Transport, Faculty of Operation and Economics of Transport and Communications, University of Zilina, Univerzitna 8215/1, 010 26 Zilina, Slovakia; milos.poliak@uniza.sk

**Keywords:** materials selection, canine vehicle restraint, expanded polypropylene, polyurethane foam, polyamide, environmental ageing, service-life prediction, crashworthiness, circular design, polymer recycling

## Abstract

Canine vehicle restraint systems are multi-material safety products in which polymer structure and condition govern energy absorption, containment, restraint, comfort, hygiene, and durability. This critical scoping review integrates materials-science studies, canine exposure studies, standards, producer data sheets, and manufacturer declarations to compare expanded polypropylene (EPP), polypropylene (PP), flexible polyurethane (PU) foams, polyamide (PA), poly(ethylene terephthalate) (PET), thermoplastic polyurethane (TPU), and glass-fibre-reinforced polymers. Quantitative comparison shows that moisture conditioning halves the yield strength of PA6, that conditioned PA6 loses 85% of its notched impact strength at −30 °C, that EPP crushing strength increases 3.45-fold between 20 and 60 kg/m^3^, and that viscoelastic PU foam hardens about 58-fold between 20 and −20 °C. Reproducible ageing-sensitivity and circularity classes, derived from explicit indicator thresholds, place PP/EPP and PET in the most favourable region at 1.3–2.1 USD/kg. A property-based service-life model with normalised margins, a hard-fail step function, and stated Arrhenius validity conditions is proposed and linked to dynamic safety. A Polymer Material Safety and Sustainability (PMSS) framework is compared with established methods and illustrated by an energy-absorber example. Matched dynamic testing of aged restraints is identified as the priority research need.

## 1. Introduction

Passenger-car transport systems for companion dogs occupy an unusual engineering space. They must function as consumer products during everyday use, as containment systems during braking and evasive manoeuvres, and as safety structures during a collision. The mechanical system may include a body-worn harness, a soft or rigid seat-mounted carrier, a cargo-area crate, or a hybrid carrier-and-base assembly. Although metal components may provide local structural load paths, polymeric materials frequently dominate the dog-facing surfaces, energy-absorbing elements, webbing, shell components, connectors, liners, coatings, and interfaces that determine how the complete system behaves.

The crashworthiness problem has previously been treated mainly at product or restraint-system level. Direct experimental work on dog travel crates has shown that apparent performance depends strongly on the test fixture and vehicle-side boundary conditions [1]. Field surveys also show that dogs are transported using heterogeneous restraint practices [2]. However, the material condition of a restraint at the time of a crash is equally important. A product that was mechanically adequate when new may no longer provide the same load distribution, impact absorption, or closure integrity after years of thermal cycling, ultraviolet (UV) exposure, moisture uptake, abrasion, repeated cleaning, cyclic compression, and animal-induced damage.

Material selection also interacts with animal welfare. Road transport can induce measurable physiological stress [3], while cage geometry affects posture, balance, and the animal’s ability to stabilise itself [4]. Vehicle interiors may experience substantial temperature excursions [5,6]. These conditions matter for polymers because elevated temperature accelerates diffusion, oxidation, and volatile release; UV radiation promotes photo-oxidation; moisture changes the response of hygroscopic polymers; and repeated dog contact introduces saliva, skin oils, claws, hair, urine, cleaning agents, and local abrasion. The relevant service environment is therefore a coupled thermal–chemical–mechanical exposure rather than a simple indoor condition.

Commercial systems illustrate the diversity of material strategies. Tavo describes a pet carrier that uses expanded polypropylene (EPP) as a lightweight energy-absorbing structure and memory foam in side-impact protection [7]. MIMsafe Care2 combines a shatter-resistant nylon (polyamide, PA) structure with aircraft-grade aluminium [8]. The 4pets PRO NOIR uses aluminium profiles and glass-fibre-reinforced polymer (GFRP) connectors and lock housings [9]. Sleepypod specifies luggage-grade ballistic nylon and a neoprene buckle shield in its Clickit Range harness [10], whereas the TRIXIE 13176 seat is a polyester-based textile product with foam padding [11]. Products sold for the same purpose may therefore depend on fundamentally different polymer families and ageing mechanisms.

The central materials-engineering question is therefore not only whether a transport system passes a crash test when new, but whether its multi-material architecture retains the required mechanical and functional properties throughout realistic service exposure while minimising chemical and particulate release and enabling responsible end-of-life management. This issue becomes especially important as the automotive sector increases recycled-polymer content and adopts design-for-circularity strategies.

No review identified in the present search integrated polymer environmental ageing, crash-critical property retention, canine contact exposure, and end-of-life design for vehicle restraint products within one materials framework. Accordingly, this review has five objectives: (i) to identify the principal polymer families used in canine vehicle restraint and transport systems and to compare them quantitatively (density, stiffness, strength, impact resistance, moisture uptake, temperature limits, and cost); (ii) to relate their structure and properties to energy absorption, containment, comfort, and vehicle attachment; (iii) to evaluate ageing mechanisms, define a property-based service-life model, and link property loss to dynamic safety performance; (iv) to examine chemical and particulate exposure of dogs and the end-of-life options for each polymer family; and (v) to propose and illustrate a Polymer Material Safety and Sustainability (PMSS) framework and a material architecture for a next-generation carrier mounted on ISOFIX (International Organization for Standardization FIX) vehicle anchorages [12]. The review is organised accordingly: Section 2 describes the review methodology and the scoring rules used for material comparison; Section 3 presents the materials, their quantitative properties and cost; Section 4 addresses environmental ageing, service-life modelling, and consequences for dynamic safety; Section 5 covers chemical and particulate exposure and circularity; Section 6 presents the PMSS framework, a worked example, the proposed architecture, and the validation programme; and Section 7 summarises the discussion, limitations, and conclusions.

## 2. Review Methodology and Evidence Framework

### 2.1. Review Design, Information Sources, and Search Strategy

The study was designed as a critical, materials-centred scoping review and reported following the applicable items of the Preferred Reporting Items for Systematic Reviews and Meta-Analyses extension for Scoping Reviews (PRISMA-ScR) [13]. A statistical meta-analysis was not attempted because the evidence base combines polymer ageing experiments, crashworthiness studies, toxicological biomonitoring, recycling research, standards, and manufacturer material declarations, which use incompatible specimen geometries, exposure conditions, and outcome metrics. The literature was identified in PubMed and in publisher and journal databases resolved through DOI/Crossref (ScienceDirect, MDPI, SpringerLink, Wiley Online Library, and Taylor & Francis Online), with searches updated through 16 September 2026, and was complemented by the official International Organization for Standardization (ISO), International Electrotechnical Commission (IEC), and ASTM International catalogues for standards, producer data sheets, and official manufacturer technical pages. To make the record counts reproducible, the database search blocks were re-executed in OpenAlex, which indexes Crossref-registered publications of all major publishers, on 22 October 2026. No lower publication-date limit was imposed because several foundational polymer ageing and recycling papers remain directly relevant to mechanism and lifetime modelling; only English-language sources were considered.

Five search blocks were used (Table 1); the strings in Table 1 are given exactly as executed, and the searched field (title, or title and abstract) is stated for each string, because broad polymer terms searched in abstracts return records dominated by unrelated applications. Reference lists of highly relevant review and experimental papers were additionally screened (backward citation chaining), and citing articles of key papers were checked (forward chaining).

### 2.2. Eligibility Criteria, Screening, and Appraisal

Sources were included when they met at least one of the following criteria: (a) experimental evidence on polymer structure, ageing, property retention, recycling, migration, emissions, or particle release; (b) peer-reviewed evidence on canine exposure or transport conditions relevant to material selection; (c) a current international standard applicable to polymer ageing, foams, textiles, or restraint testing; (d) an official manufacturer declaration or technical data sheet identifying a material or property used in this review. Duplicate records, non-authoritative retail or affiliate pages, unsourced toxicity claims, and product pages that did not identify a material or relevant engineering feature were excluded. Manufacturer information was used to document material architecture and typical property values only and was not treated as independent evidence of durability, toxicological safety, or comparative superiority.

The database searches returned 6123 records in total (sum of blocks A–D, without removal of overlaps between blocks; Table 1). Records were screened by title and abstract against the eligibility criteria, potentially relevant records were assessed in full text, and uncertainties were resolved by discussion among the authors. Together with sources obtained by citation chaining and by targeted retrieval of standards, data sheets, market data, and product declarations, 92 sources were included. Each included source was appraised for relevance using three binary questions: whether it addresses one of the seven material families considered (EPP/polypropylene (PP), polyurethane (PU) foam, PA, poly(ethylene terephthalate) (PET), thermoplastic elastomers (TPE) or thermoplastic polyurethane (TPU), and GFRP); whether it reports quantitative data or a mechanistic explanation; and whether its exposure or loading conditions are representative of a vehicle cabin. Full texts were excluded when they were outside the material scope, contained no quantitative or mechanistic content, or came from a non-authoritative or unverifiable source.

### 2.3. Evidence Hierarchy

Evidence was interpreted using four levels. Level I comprised peer-reviewed experimental studies directly characterising polymer properties, ageing, recycling, chemical migration, or canine exposure. Level II comprised peer-reviewed reviews, handbooks, and international standards used to establish general mechanisms, property ranges, and test methods. Level III comprised official manufacturer documentation and producer data sheets specifying material composition, typical properties, or declared certification. Level IV comprised engineering inference where a material family could be identified but grade, additive package, or processing history was not disclosed. Level IV information was used only to formulate research needs and hypotheses, not to claim product-specific toxicity or lifetime; where it is used in the text, it is explicitly identified as a hypothesis.

### 2.4. Assessment Domains and Transparent Scoring Rules

The material assessment was organised around seven interacting domains: structural and energy-management function; environmental ageing; mechanical damage and fatigue; hygiene and animal-contact exposure; chemical transparency and additive burden; repairability and replacement of sacrificial components; and recyclability and end-of-life separation. A material is considered suitable only if it simultaneously satisfies its mechanical function and remains acceptably stable during the intended service environment.

To make the comparative figures reproducible, two ordinal indices were defined: an ageing-sensitivity class and a circularity class. Each index is derived from four quantitative or verifiable indicators. Each indicator is converted into a sub-score of 1, 3, or 5 using explicit thresholds (Section 4.3 and Section 5.3), and the class is the arithmetic mean of the determined sub-scores, rounded to the nearest integer; materials with fewer than two determined indicators are not classified. The indicator values and their sources are reported for every material, so that any reader can recompute or contest a score. Because the classes are ordinal, they are used only to order the material families and are not interpreted as measured quantities. A single cross-material “crash-function suitability” score used in an earlier version of this analysis was abandoned because the materials serve different functions (energy absorption, tensile restraint, structural containment) that cannot be placed on one scale; function-specific quantitative properties are compared instead (Section 3.2).

## 3. Polymer Materials: Properties, Cost, and Functional Roles

### 3.1. Material Architectures of Representative Commercial Systems

Commercial disclosure is uneven. Some manufacturers identify polymer families such as EPP, nylon, polyester, or glass-fibre-reinforced plastic, whereas others describe only generic foam, fabric, or plastic. This disclosure gap is relevant to service-life and circularity assessment because unknown resin grades and additive packages prevent robust prediction of UV stability, thermal oxidation, chemical migration, and recycling compatibility. Table 2 summarises representative systems for which material information could be verified from official sources and identifies, for each, the dominant ageing concern and the validation information still missing. The objective is not to rank brands: public declarations are incomplete and no independent comparative ageing programme was identified.

### 3.2. Quantitative Property and Cost Comparison

Table 3 and Table 4 compile typical property values and indicative prices for the material families considered, including PP and PA6 reinforced with 30 wt% short glass fibres (PP-GF30 and PA6-GF30) as representatives of GFRP. To keep the comparison verifiable, each row refers to an identified commercial grade or to a cited study, rather than to undocumented generic ranges; individual grades can differ substantially, and the values are intended to show orders of magnitude and directions of change, not design allowables. Prices are indicative European market values for 2025–2026 reported by market-price services, given in the currency of the source; they fluctuate with feedstock prices and should be re-checked at the time of design.

**Figure 1:**
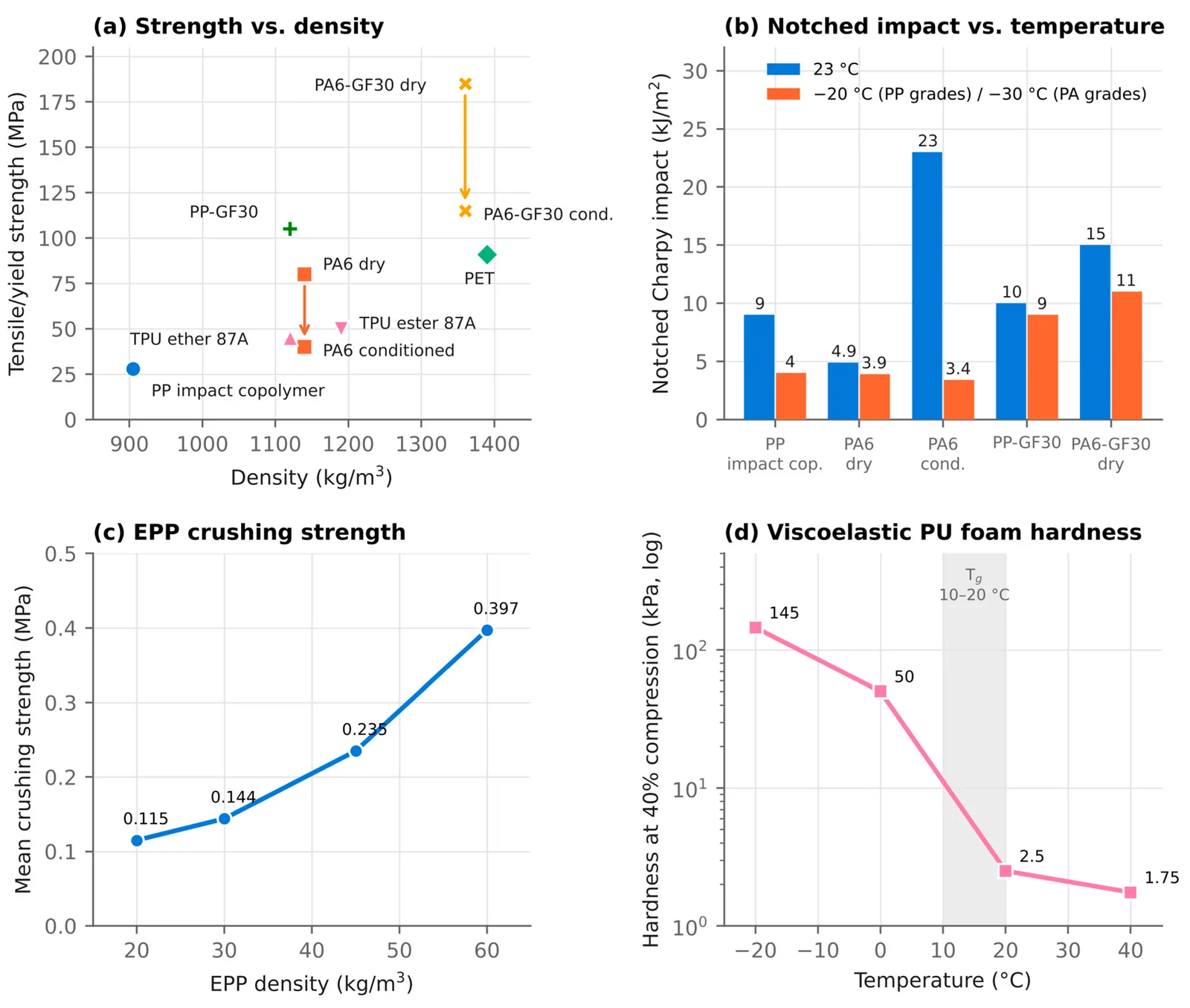
Quantitative comparison of the principal material families. (**a**) Tensile or yield strength versus density of solid polymers and short-glass-fibre compounds; arrows show the effect of moisture conditioning (dry → 23 °C/50% RH) on PA6 and PA6-GF30 [14,18,19,21,23,25,37]. (**b**) Notched Charpy impact strength at 23 °C and at −20 °C (PP grades) or −30 °C (PA grades) [14,18,23,25]. (**c**) Mean crushing strength of EPP versus density, quasi-static compression at 12 mm/min [28]. (**d**) Hardness at 40% compression of a viscoelastic PU foam versus temperature (logarithmic scale); the shaded band marks the glass transition (10–20 °C) [33]. Values are for the identified grades and are not design allowables.

Figure 1 visualises the data that are most relevant to the safety function. Three observations follow directly. First, moisture conditioning halves the yield strength of PA6 (80 → 40 MPa) and reduces the strength of PA6-GF30 by 38% (185 → 115 MPa), whereas PP and PET absorb no more than 0.03% water (Figure 1a). Second, conditioned PA6 is tough at 23 °C (23 kJ/m^2^) but loses 85% of its notched impact strength at −30 °C (3.4 kJ/m^2^), and the PP impact copolymer loses 56% between 23 and −20 °C, while PP-GF30 retains 90% (Figure 1b); winter qualification is therefore not optional for PP and PA latches, housings, and connectors. Third, the cellular materials differ by orders of magnitude in their temperature stability: the crushing strength of EPP is set mainly by density, increasing 3.45-fold between 20 and 60 kg/m^3^ [28] (Figure 1c), whereas the hardness of a viscoelastic PU foam increases about 58-fold between 20 and −20 °C because its glass transition lies at 10–20 °C [33] (Figure 1d). On a cost basis, PP, PET, and PP-GF30 are the least expensive structural candidates (1.3–2.1 USD/kg), PA6 is moderately more expensive (1.95–2.12 USD/kg), and TPE/TPU is the most expensive of the thermoplastics considered (≈2.6 USD/kg); recycled PP regranulate was quoted at 0.77–0.78 €/kg in early 2026 [17].

### 3.3. Material Families: Structure, Function, and Limitations

#### 3.3.1. Polyolefins: EPP Foam and Impact-Modified PP

EPP is attractive for vehicle-facing energy-management components because it combines low density, recoverable deformation over moderate strains, chemical resistance, and a tunable crushing plateau, and it is already widely used in automotive impact-management applications [40]. Compression studies show that density strongly affects crushing strength, energy absorption, and specific energy absorption [28]: increasing EPP density from 20 to 60 kg/m^3^ increased the mean crushing strength by 245.2% (0.115 → 0.397 MPa), total energy absorption by 187.2% (54.06 → 155.49 J), energy absorption efficiency by 42.3%, specific energy absorption by 54.3%, and energy absorbed per unit volume by 242.8% (Figure 1c). Density, thickness, bead-fusion quality, and available crush stroke must therefore be specified jointly as safety-critical parameters rather than as generic foam characteristics.

Impact-modified PP is a candidate for injection-moulded housings because of its low density and established recycling infrastructure (Table 3). The main durability concern for both forms is oxidation: polyolefins undergo thermo-oxidative ageing in which stabilisers are progressively consumed, oxygen-containing groups accumulate, and chain scission ultimately reduces molecular weight and toughness [41]. UV exposure is a separate qualification variable, because it produces surface-concentrated photo-oxidation [42]. A PP or EPP component should therefore be assessed through retained force–displacement response and impact-energy absorption, not visual appearance.

#### 3.3.2. Flexible PU and Viscoelastic Foams

Flexible PU foams and viscoelastic (memory) foams are useful where local cushioning, side-impact contact management, pressure distribution, or comfort are required, and their chemistry can be tailored over a broad range of stiffness and damping. Flexible PU is a crosslinked network, so it cannot be remelted, and ageing may include oxidation, hydrolysis of susceptible linkages, compression set, embrittlement, and changes in cell structure; the ageing behaviour depends on hard-segment structure and phase separation, so the generic label “PU foam” is insufficient for service-life prediction [43]. Oxidative ageing is also relevant to in-vehicle air quality, because autoxidation of flexible PU foam can generate carbonyl VOCs including formaldehyde, acetaldehyde, and acrolein, at rates strongly dependent on temperature and formulation [44]. In a canine carrier, PU inserts should therefore be removable and replaceable rather than bonded into a mixed-material assembly.

#### 3.3.3. Polyamides and Ballistic Nylon

Polyamides are used in high-strength technical textiles, injection-moulded components, and rigid or semi-rigid carrier structures because of their strength, fatigue and abrasion resistance, and toughness. Their main limitation is moisture sensitivity: water uptake disrupts inter-chain hydrogen bonding and plasticizes the polymer, reducing stiffness and strength while increasing ductility (Table 3), and UV exposure can promote photo-oxidation and embrittlement [45]. For harnesses and carrier structures, tensile strength measured on dry, new material is therefore not sufficient; qualification should use conditioned specimens, wet/dry cycling, UV exposure, and abrasion at belt-routing regions.

#### 3.3.4. PET Textiles and Webbing

Polyester, most commonly PET, is widely used in seat fabrics, covers, straps, and technical textiles because of its dimensional stability, low water uptake relative to polyamides, washability, and established fibre-processing routes. Repeated washing, flexing, claw abrasion, and UV exposure can nevertheless damage fibres and increase microfibre release [46,47,48]. Dog-facing textiles should therefore be assessed for tensile performance, cleanability, abrasion resistance, and shedding after ageing rather than only when new.

#### 3.3.5. Elastomers, TPE/TPU, and Neoprene

Elastomeric materials appear in buckle shields, anti-slip feet, seals, pads, and local dampers. Neoprene (polychloroprene) offers cushioning and resistance to water and many chemicals, but as a crosslinked elastomer it is difficult to remelt and recycle mechanically. TPE and TPU grades provide similar functional compliance while enabling thermoplastic processing. Their relevant ageing modes are hardening, softening, permanent set, surface cracking, and loss of damping; because elastomer response is strongly temperature-dependent, temperature-conditioned dynamic characterisation is necessary [49].

#### 3.3.6. Fibre-Reinforced Polymer Composites

GFRPs are attractive for connectors and load-bearing frames because they provide high specific stiffness and strength (Table 3). Their performance depends on fibre orientation and length, matrix chemistry, processing defects, moisture, and interface integrity, and ageing can produce matrix microcracking, interfacial debonding, and local stiffness loss without visible change in geometry. Mechanical recycling shortens fibres, whereas thermal and solvolytic fibre-recovery routes require more complex processing [50]. Composite use should therefore be restricted to locations where its mechanical benefit is clear and the component can be separated.

#### 3.3.7. Coatings, Adhesives, and Functional Additives

Water-repellent, stain-resistant, antimicrobial, flame-resistant, UV-stabilised, and odour-control finishes can improve service performance but introduce a second layer of chemistry that is often invisible in product descriptions; research increasingly emphasises lower-impact and non-fluorinated water-repellent strategies [51]. Permanent adhesive bonding between foam, textile, composite, and metal components may improve local stiffness but makes end-of-life separation difficult; mechanical fastening, reversible attachment, replaceable liners, and material identification should be preferred where crash load paths permit.

### 3.4. Structure–Property–Function Relationships

Material choice must be linked to the function of each component, because energy absorption, tensile restraint, structural containment, side-contact cushioning, anti-slip behaviour, and washable comfort cannot be optimised with one property. Table 5 relates the structural basis of each family to its candidate functions. For crash-critical parts, property retention is more important than nominal virgin-material performance: a higher initial modulus is not beneficial if ageing creates brittle failure, and a very compliant foam is not safer if densification occurs too early or residual deformation allows excessive carrier excursion. Animal welfare adds further constraints: dog-facing surfaces should avoid sharp fragments, excessive heat storage, abrasive fibre ends, and poorly ventilated closed-cell assemblies, and components that can be bitten or licked require attention because animal behaviour creates oral exposure pathways uncommon for automotive trim parts.

## 4. Environmental Ageing, Service-Life Modelling, and Dynamic Safety

### 4.1. Vehicle-Cabin Service Environment

A canine transport system experiences a demanding microenvironment. In parked cars on clear days, cabin air temperatures of 43–62 °C were measured by 13:00 at ambient temperatures of 15–34 °C, with a largest 60 min rise of 25.8 °C [6]; after about one hour in the sun, mean surface temperatures reached 69 °C on the dashboard and 51 °C on the seat, against 48 and 41 °C in shade [52]. A two-year monitoring study of four UK cars recorded cabin temperatures from −7.4 to 54.5 °C, with values above 35 °C occurring from April to September [5]. A qualification envelope of at least −30 to +70 °C for dog-facing and structural polymer parts is therefore justified, and the upper bound lies above the glass transition of PA6 (45 °C) and of viscoelastic PU foam (10–20 °C) but below that of PET (81 °C) (Table 3 and Table 4). In addition, oxygen is continuously available; UV radiation reaches exposed components through glazing; wet fur, saliva, urine, cleaning water, and disinfectants introduce moisture and chemicals; and the dog produces repeated local loads by sitting, standing, scratching, and chewing.

### 4.2. Degradation Mechanisms and Quantitative Evidence

Oxidation is the central long-term degradation mechanism of most of the polymers considered, and its relationship with property loss and lifetime prediction is well established [53]. In polyolefins, thermal oxidation can initially be masked by crystallinity changes before antioxidant depletion and chain scission produce a marked reduction in toughness: in oven ageing at 115–135 °C, tensile strength peaked after 29 days at 125 °C and was significantly lower after 98 days, while the surface oxygen-to-carbon ratio fell from 0.195 to 0.135 after 29 days and 0.128 after 98 days [41]. UV exposure adds surface-concentrated photo-oxidation; for post-consumer recycled PP exposed to 225–1240 kJ/m^2^ (at 340 nm), an interior-grade compound retained stable tensile strength and showed only a small decrease in impact energy [42]. Thermal ageing and UV exposure should, therefore, be qualified separately rather than treated as interchangeable.

Moisture is the dominant variable for polyamides. A 3D-printed PA6 composite gained 5.6 ± 0.48% mass after 504 h at 50% relative humidity (RH), and its Charpy impact energy decreased from 0.463 to 0.28 J/mm^2^ (−40%); UV-C exposure reduced it to 0.32 J/mm^2^ (−31%) while increasing tensile strength from 29 to 33.6 MPa and Shore D hardness from 59.1 to 66.8, i.e., the material became stiffer but less tough [45]. For nylon 6,6 webbing, 15 days of xenon-arc exposure (stated to correspond to 107 days of outdoor exposure in Arizona) reduced breaking strength by 20% for coloured webbings and by 6% for white webbing [54], showing that colourant and stabiliser package are as important as polymer family. Dog-facing components also encounter saliva (salts, proteins, enzymes) and cleaning agents (surfactants, alcohols, oxidants), so compatibility testing should include realistic cleaning cycles and synthetic canine-saliva extraction.

Mechanical degradation is component-specific. Foams develop permanent set under repeated or sustained compression: for TPU, the most pronounced increase in compression set occurred between 70 and 80 °C, and water increased compression set through plasticization [49]; data-sheet values for 87 Shore A TPU are 25% at 23 °C but 35–45% at 70 °C (Table 4). Textiles shed fibres under abrasion: after 5000 Martindale rubs, 12 polyester textiles released 4900–640,000 microplastic fibres and up to 350,000 fibrils per gram, more than ten times the release caused by washing alone [47]. Moulded connectors experience cyclic bending and notch-sensitive crack initiation, and composite joints accumulate damage at holes and rivets; these mechanisms can occur without obvious gross failure.

### 4.3. Ageing-Sensitivity Classification

**Figure 2:**
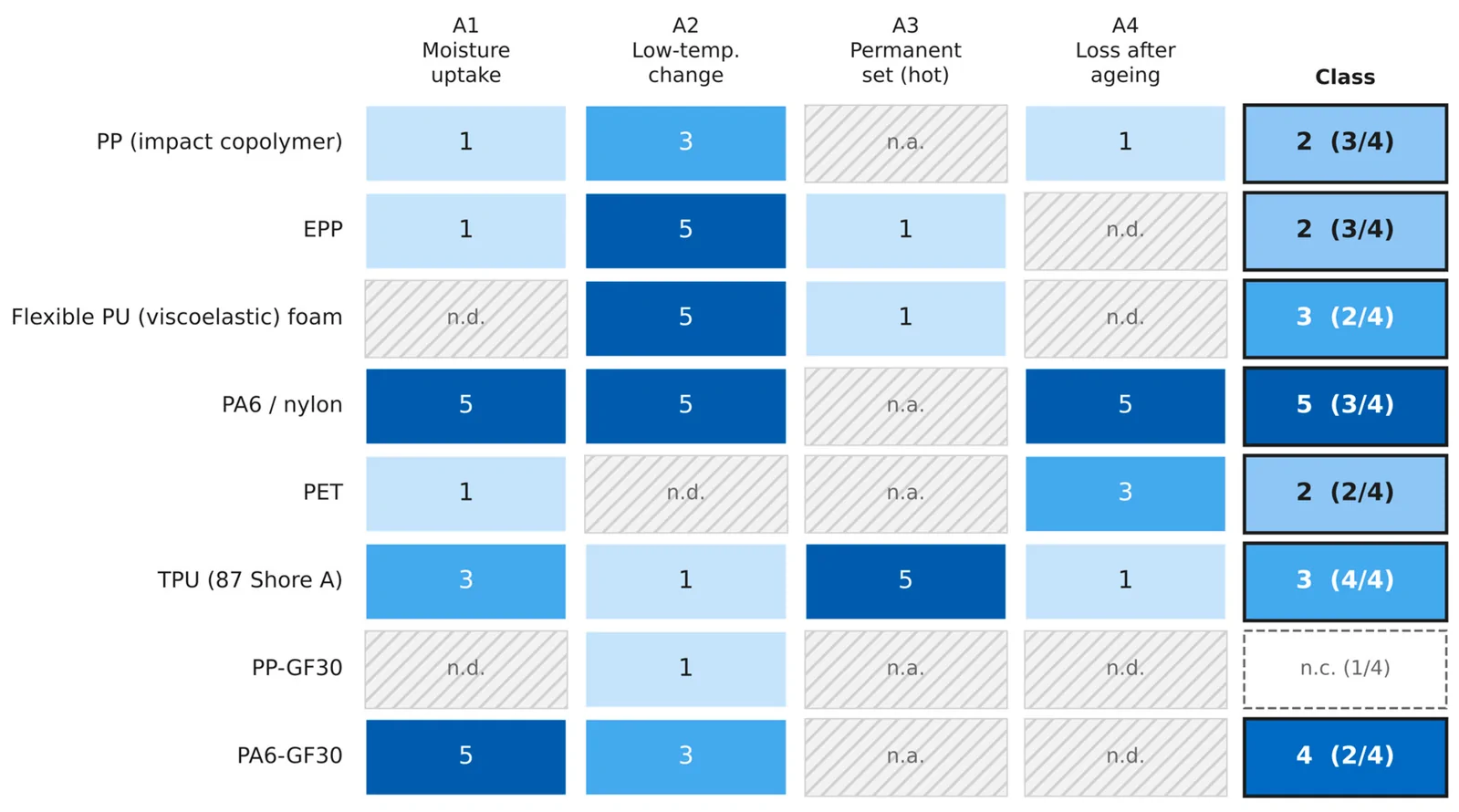
Ageing-sensitivity sub-scores (A1–A4) and resulting class for the principal material families, derived from the indicator values and thresholds in Table 6 and Table 7. Sub-score 1 = low, 2 = low-to-moderate, 3 = moderate, 4 = moderate-to-high, 5 = high sensitivity; class = rounded mean of determined sub-scores; (k/4) = number of indicators determined; n.d. = not determined; n.a. = not applicable; n.c. = not classified.

To replace the earlier author-assigned scores with a reproducible classification, ageing sensitivity is rated by four indicators (Table 6): moisture uptake (A1), change of the safety-relevant property between 23 °C and −20/−30 °C (A2), permanent set at elevated temperature for cellular and elastomeric materials (A3), and the largest documented property loss after accelerated ageing (A4). Each indicator value is converted to a sub-score of 1, 3, or 5 using the thresholds in Table 6; the class is the rounded mean of the determined sub-scores, and materials with fewer than two determined indicators are not classified. Table 7 lists every indicator value with its source, and Figure 2 shows the resulting sub-scores and classes.

The classification separates three groups. PA6 (class 5) and PA6-GF30 (class 4) are the most ageing-sensitive because moisture uptake and low-temperature embrittlement act together. Flexible PU foam and TPU fall into class 3, but for different reasons: the viscoelastic foam because of its extreme temperature dependence near its glass transition, TPU because of high compression set at 70 °C. PP, EPP, and PET fall into class 2; for EPP, this class conceals one high sub-score (temperature dependence of collapse stress), which is why the energy absorber must still be qualified at −30 and +70 °C (Section 4.4). PP-GF30 is not classified because only one indicator could be determined. Evidence coverage is reported with every class because classes based on two indicators are less robust than those based on four.

### 4.4. Consequences of Ageing for Dynamic Safety

The safety relevance of ageing lies in how property changes alter the structural response of the restraint during a collision or sudden deceleration. Four links can be identified, and Table 8 distinguishes the part of each link that is experimentally demonstrated from the part that is an engineering hypothesis requiring system-level testing.

First, loss of available crush stroke in an energy absorber raises the deceleration of the protected mass. If a crushable element with an approximately constant plateau stress must dissipate the relative kinetic energy of the dog and carrier over a stroke s, the mean deceleration and its change after a permanent stroke loss δ (as a fraction of the original stroke) are approximately.

(1) $${\bar{a}}_{\mathrm{mean}}\;\approx \;\frac{{v_{\mathrm{rel}}}^{2}}{2\;s},\;\frac{{\bar{a}}_{\mathrm{mean},\mathrm{aged}}}{{\bar{a}}_{\mathrm{mean},\mathrm{new}}}=\frac{s_{\mathrm{new}}}{s_{\mathrm{aged}}}=\frac{1}{1-\delta }$$

where ā_mean_ is the mean deceleration during crushing; v_rel_ is the relative velocity to be dissipated; s is the available crush stroke before densification; and δ is the fractional stroke loss caused by compression set, residual crush, or prior impact. Equation (1) is a first-order energy balance and neglects rate effects and elastic rebound.

A stroke loss of 10% therefore raises the mean deceleration by 11%, and a loss of 20% by 25%; if the residual stroke becomes shorter than required, the absorber densifies and the peak deceleration rises sharply. Second, a change in plateau stress shifts the force level directly: a collapse-stress increase of about 110% at −30 °C [31,32] would roughly double the force transmitted by an EPP absorber of fixed area, whereas a 50% decrease at 60 °C halves the energy that can be dissipated within the same stroke. Third, embrittlement changes the failure mode of load-path components: conditioned PA6 retains only 15% of its notched impact strength at −30 °C, and the impact energy of a PA6 composite decreased by 40% after humidity exposure [18,45]; a latch or connector that deforms ductilely when new may fracture when cold or aged. Fourth, strength loss of webbing reduces the safety margin of the tensile restraint path, as shown by the 6–20% reduction in breaking strength of nylon webbing after accelerated UV exposure [54].

System-level evidence for these links remains limited and partly reassuring. In sled tests of child restraint systems aged from 3 to 269 months, no clear relationship between dummy responses and seat age was found, although the head excursion of forward-facing seats increased by 0.27 mm per month of age (95% confidence interval 0.13–0.42; R^2^ = 0.59) and the odds of critical damage increased by a factor of 1.031 per month [56]. Field-used expanded-polystyrene bicycle helmets up to 26 years old showed no loss of impact attenuation, with an age effect of at most 0.76 g per year [57]. These findings indicate that well-stabilised foam liners can retain their function for long periods, but that ageing of plastic shells, connectors, and textiles can produce measurable kinematic drift. Equivalent data for canine restraints do not exist.

**Table 8:** Links between ageing-induced material changes and dynamic safety performance, with evidence status.

Material Change	Quantitative Material Evidence	Expected Effect on Dynamic Response	Evidence Status
Permanent set/residual crush of EPP or PU absorber	EPP compression set 14–16% after 30 min, 3–4% after 24 h (25% strain, 22 h) [29,30]; TPU 35–45% at 70 °C [37]	Mean deceleration rises by 1/(1 − δ) (Equation (1)); risk of densification	Material: demonstrated. Effect on system: first-order mechanics; hypothesis to be confirmed by matched sled tests
Temperature shift in foam collapse stress or hardness	EPP +110% (−30 °C), −50% (60 °C) [31,32]; viscoelastic PU hardness ×58 between 20 and −20 °C [33]	Cold: higher transmitted force; hot: lower energy capacity, bottoming out	Material: demonstrated. System: hypothesis
Moisture- and cold-induced embrittlement of PA	Conditioned PA6 notched impact 23 → 3.4 kJ/m^2^ at −30 °C [18]; −40% impact after 504 h at 50% RH [45]	Change from ductile deformation to fracture of latches, hinges, connectors	Material: demonstrated. System: hypothesis
UV strength loss of webbing	Nylon 6,6 webbing −6 to −20% breaking strength [54]	Reduced margin against restraint rupture; higher elongation and excursion	Material: demonstrated. System: hypothesis
Recycled-content contamination in PP-GF	Impact strength 15.2 → 8.2 kJ/m^2^ at 70% rPP [58]	Lower fracture resistance of structural parts	Material: demonstrated. System: hypothesis
Long-term ageing of complete restraints	Child restraints: head excursion +0.27 mm/month; odds of critical damage ×1.031/month [56]; helmets: ≤0.76 g/year [57]	Small but measurable drift of kinematics	System-level: demonstrated for child restraints and helmets; not for canine products

Table 9 translates these links into a failure-mode matrix that can be used in design reviews and maintenance instructions.

### 4.5. Property-Based Service-Life Model

Universal statements such as “nylon lasts five years” or “EPP lasts ten years” are not defensible for safety-critical products, because service life depends on resin grade, stabiliser package, geometry, manufacturing history, exposure, and load history. Service life is therefore defined here through safety-critical properties, with an explicit distinction between gradual performance loss and irreversible critical events. The descriptive retention of property i is

(2) $$R_{i}\left(t\right)=\frac{P_{i}\left(t\right)}{P_{i,0}}$$

where P_i_(t) is the measured value of safety-critical property i after service or accelerated-ageing time t, and P_i,0_ is the mean value of the qualified unaged batch. R_i_ is a descriptive quantity; it is not used directly for decisions because the acceptable retention differs between properties.

For decisions, each property is normalised by its own acceptance limit, giving a dimensionless margin that equals 1 for the new material and 0 at the acceptance limit:

(3) $$M_{i}\left(t\right)\;=\;\frac{P_{i}\left(t\right)\;-\;P_{i,\min}}{P_{i,0}\;-\;P_{i,\min}},\;\mathrm{for}\;\mathrm{properties}\;\mathrm{where}\;\mathrm{larger}\;\mathrm{is}\;\mathrm{better}$$

(4) $$M_{i}\left(t\right)=\frac{P_{i,\max}-P_{i}\left(t\right)}{P_{i,\max}-P_{i,0}},\;\mathrm{for}\;\mathrm{properties}\;\mathrm{where}\;\mathrm{smaller}\;\mathrm{is}\;\mathrm{better}$$

where M_i_(t) is the normalised margin of property i; P_i,min_ is the minimum acceptable value of a property for which larger is better (e.g., impact strength, absorbed energy, breaking force); and P_i,max_ is the maximum acceptable value of a property for which smaller is better (e.g., compression set, residual crush, migration or emission rate). The normalisation basis is therefore the distance between the qualified new state and the acceptance limit, which makes margins of different properties comparable regardless of their units.

The acceptance limits are determined experimentally rather than assumed. Specimens are conditioned to several degradation levels, the property is measured on coupons, and matched complete systems with the same conditioning are tested dynamically (Section 6.5). The limit is the lower statistical tolerance bound of the property values for which the system still met its dynamic acceptance criteria (e.g., carrier excursion, peak acceleration, absence of structural fracture):

(5) $$P_{i,\min}\;=\;{\bar{P}}_{i,\mathrm{pass}}\;-\;k_{n,0.95,0.95}\;s_{i,\mathrm{pass}}$$

where ${\bar{P}}_{i,\mathrm{pass}}$ and s_i,pass_ are the mean and standard deviation of property i in the lowest conditioning level that still passed the dynamic test (n specimens), and k_n,0.95,0.95_ is the one-sided normal tolerance factor for 95% coverage and 95% confidence (4.20 for *n* = 5; 2.91 for *n* = 10). The analogous upper bound is used for P_i,max_.

Gradual performance loss ends service when the first margin reaches zero, whereas critical events act as an irreversible step that is not captured by gradual retention:

(6) $$t_{G}\;=\;\underset{i}{\min}\inf\left\{t\;:\;M_{i}\left(t\right)\;\leq \;0\right\}$$

(7) $$H\left(t\right)=\left\{\begin{array}{c} 0,\;t<t_{H}\\ 1,\;t\;\geq \;t_{H} \end{array}\right.,\;t_{\mathrm{SL}}=\min\left(t_{G},\;t_{H}\right)$$

where t_G_ is the gradual-loss limit; H(t) is a step (Heaviside) function that switches irreversibly from 0 to 1 at time t_H_, the first occurrence of a non-recoverable damage state (crash activation of an absorber, cracked connector, torn restraint, delaminated composite, closure malfunction); and t_SL_ is the service-life limit. Because t_H_ is a random event time rather than a degradation trend, it is controlled by inspection and post-crash replacement rules, whereas t_G_ is predicted from ageing data.

For the complete system, the conservative condition is

(8) $$M_{\mathrm{sys}}\left(t\right)=\left[1-H\left(t\right)\right]\;\cdot \;\underset{i\;\in \;\left\{S,\;E,\;T,\;C\right\}}{\min}M_{i}\left(t\right)$$

where M_sys_(t) is the system margin, and the index i covers the structural (S), energy absorption (E), tensile restraint (T), and closure/connector (C) functions. The system is serviceable while M_sys_(t) > 0. Unlike a plain minimum of retention ratios, this formulation is normalised against validated acceptance limits and becomes zero immediately when a hard-fail event occurs, irrespective of the remaining margins.

Table 10 lists the critical properties, hard replacement triggers, and recommended actions for the principal polymer components. Calendar age remains useful for scheduling inspections, but replacement should be tied to the margins and hard-fail states defined above, and manufacturers should publish the qualified ageing envelope, inspection criteria, and post-crash replacement rules.

### 4.6. Accelerated Ageing and Validity of Arrhenius Extrapolation

Accelerated ageing is necessary because calendar-life testing is impractical, but the elevated test temperature must not create a degradation mechanism that does not occur in service. For a degradation process with a single dominant mechanism, the time-scaling between test and use temperature can be described by the Arrhenius acceleration factor (AF) [53]:

(9) $$AF\;=\;\frac{t_{\mathrm{use}}}{t_{\mathrm{test}}}\;=\;\exp\left[\frac{E_{a}}{R}\left(\frac{1}{T_{\mathrm{use}}}\;-\;\frac{1}{T_{\mathrm{test}}}\right)\right]$$

where AF is the acceleration factor; t_use_ and t_test_ are the times needed to reach the same value of the property under use and test conditions; E_a_ is the apparent activation energy of the governing process (J/mol); R = 8.314 J·mol^−1^·K^−1^; and T_use_ and T_test_ are absolute temperatures (K).

The sensitivity of extrapolation to E_a_ is large. For the formation of one oxidation product of flexible PU foam, E_a_ increased from 87 kJ/mol in low-index to 108 kJ/mol in high-index formulations [44]. For T_use_ = 40 °C and T_test_ = 80 °C, these values give AF = 44 and 110, respectively, so that 1000 h of testing would represent about 5 or 12.5 years of continuous exposure at 40 °C; a 21 kJ/mol uncertainty in E_a_ thus changes the predicted life by a factor of 2.5. The same study observed deviations from Arrhenius behaviour above 100 °C for formaldehyde and acrolein formation [44].

Equation (9) is therefore applied only when the following conditions are verified: (i) the property–time curves at no fewer than three test temperatures superimpose by time–temperature shifting, and ln(t_test_) is linear in 1/T_test_ without curvature; (ii) no thermal transition lies between T_use_ and T_test_, which excludes extrapolation across the glass transition of PA6 (45 °C), PET (81 °C), or viscoelastic PU foam (10–20 °C) and requires T_test_ to remain well below the onset of melting; (iii) oxidation is homogeneous through the thickness, i.e., the specimen is thinner than the diffusion-limited-oxidation depth, which is checked by oxidation profiles across the section [53]; (iv) stabilisers are not lost by evaporation or migration at T_test_ at a rate that does not occur in service; (v) humidity is held constant, because moisture-driven processes in PA and polyester-type PU require a separate humidity term; and (vi) the same failure metric is used at all temperatures, since oxidation induction time, tensile strength, impact toughness, compression set, and VOC generation can have different activation energies. Extrapolated lives must finally be verified against at least one field-aged or naturally weathered condition.

## 5. Chemical and Particulate Exposure, End-of-Life, and Circularity

### 5.1. Dog-Specific Exposure Pathways and Chemical Classes

The chemical-safety discussion requires careful source attribution. Dogs are exposed to plastic-associated chemicals and polymer-derived particles in the home environment, but available biomonitoring does not establish canine car seats, harnesses, or crates as the source. The interpretation adopted here is therefore hazard- and pathway-based: identify chemicals that can occur in relevant polymer systems, identify dog-specific contact pathways, and test migration from the actual transport product before making product-specific health claims. Dogs have several pathways that differ from those of an adult human occupant: prolonged dermal and fur contact, inhalation in a small enclosure, licking, chewing and mouthing of edges or straps, ingestion of abraded fibres or fragments, and transfer of residues from coat and paws.

Direct canine-product evidence exists for plastic toys and training devices: Wooten and Smith showed that selected phthalates and bisphenol A (BPA) leached from dog products into synthetic canine saliva and that simulated ageing or chewing influenced release for some compounds [60]. Biomonitoring confirms that pet dogs are exposed to phthalates, with multiple urinary phthalate metabolites detected [61], and reviews of endocrine-disrupting chemicals (EDCs) in companion animals identify ingestion, inhalation, and transdermal uptake as relevant pathways for phthalates, bisphenols, flame retardants, and per- and polyfluoroalkyl substances (PFAS) [62]. Dog biomonitoring also confirms exposure to organophosphate flame retardants, although food was an important pathway [63]. The preferred design approach is therefore not to assume that any additive chemistry is unsafe, but to require full material declaration, low-migration formulations, and emission and migration testing for dog-facing components.

Elevated cabin temperature increases emissions from plastics, composites, foams, rubbers, sealants, and adhesives. Automotive material testing using thermal-desorption and headspace gas chromatography–mass spectrometry (TD-GC-MS and HS-GC-MS) found particularly high total VOC potential for some composite and PU-foam materials and a strong temperature dependence of emissions [64], and kinetic work on flexible PU foams confirms that oxidation generates low-molecular-weight carbonyl VOCs [44]. Synthetic textiles release fibrous microplastics during production, abrasion, laundering, and ageing [46,47]; PET and polycarbonate (PC) microplastics have been measured in pet food and faeces, and dietary intake did not fully explain faecal levels [65]. Table 11 summarises the exposure classes and the corresponding evidence gaps for transport systems.

### 5.2. End-of-Life Routes by Material Family

Circularity is governed less by the theoretical recyclability of an individual polymer than by the architecture of the assembled product. Sorting, contamination, polymer compatibility, and degradation during reprocessing are the main barriers to mechanical recycling [66], while chemical routes can complement it for difficult waste streams [67]. The current scale of recovery is modest: in 2022 the post-consumer recycling rate of plastics in the EU27, Norway, Switzerland, and the UK was 26.9% (8.7 Mt), recycled post-consumer material covered 12.6% of new plastic products, and chemical recycling accounted for 0.1%; in the automotive sector, 18.8% of the 1.6 Mt of collected plastic waste was recycled and recycled content was 4.6% [68]. The situation differs strongly between families (Table 12). PET has the most developed loop: the collection rate of PET bottles in Europe was 60% in 2022 [69], and glycolysis and methanolysis operate at commercial scale, e.g., a 10 kt/yr glycolysis plant since 2019 [67,70]. PA6 can be depolymerized to caprolactam at industrial scale; a plant in Ljubljana processes 28 t of PA6 waste per day [71]. Flexible PU foam is crosslinked and cannot be remelted; in 2017 only 14% of the approximately 40 million mattresses reaching end of life annually in the EU were recycled, about half being landfilled [72], and glycolysis can recover polyol (about 70% within 25 min with catalysis) that replaces up to 50% of virgin polyol without significant loss of foam quality [73]. For glass-fibre-reinforced polymers, thermal and chemical fibre-recovery processes are not close to commercialization [74], mechanical recycling shortens fibres [50], and the most mature route remains grinding [75].

### 5.3. Circularity Classification

**Figure 3:**
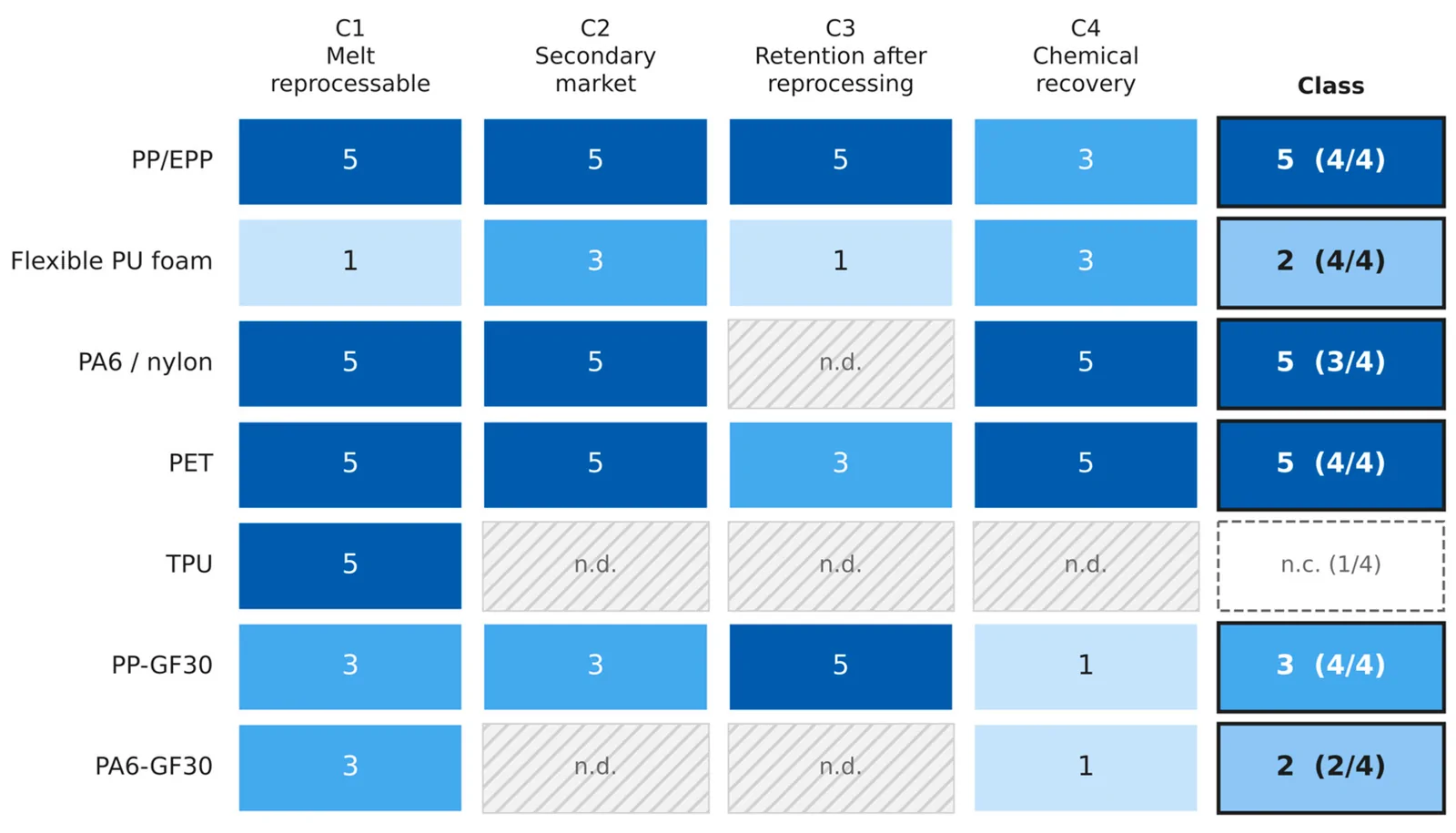
Circularity sub-scores (C1–C4) and resulting class for the principal material families, derived from the indicator values and thresholds in Table 13 and Table 14. Sub-score 1 = low, 2 = low-to-moderate, 3 = moderate, 4 = moderate-to-high, and 5 = high circularity potential; class = rounded mean of determined sub-scores; (k/4) = number of indicators determined; n.d. = not determined; n.c. = not classified.

Circularity potential is rated by four indicators (Table 13): melt reprocessability (C1), existence of a secondary-material market with a published regranulate price or documented recycling stream (C2), retention of mechanical properties after one reprocessing cycle (C3), and maturity of chemical or monomer recovery (C4). The scoring rule is the same as for the ageing class (Section 2.4). Table 14 lists the values and sources, and Figure 3 shows the resulting sub-scores. The classification refers to the material family in a clean, separable form; coatings, laminates, blends, and adhesives in a real product reduce the achievable circularity and are addressed by the design measures in Table 12 and Section 5.5.

PP/EPP and PET reach class 5 because they are thermoplastics with traded recyclates, good retention after reprocessing, and at least partial chemical routes; PA6 also reaches class 5 at material level because of industrial depolymerization, although coated technical fabrics and mixed textile constructions in real products are much harder to recover. Flexible PU foam (class 2) and PA6-GF30 (class 2) are the least circular, and PP-GF30 is intermediate (class 3) because its thermoplastic matrix can be reprocessed with only slight property loss [78], but the fibres cannot be recovered at full value. TPU could not be classified because only one indicator was documented.

**Figure 4:**
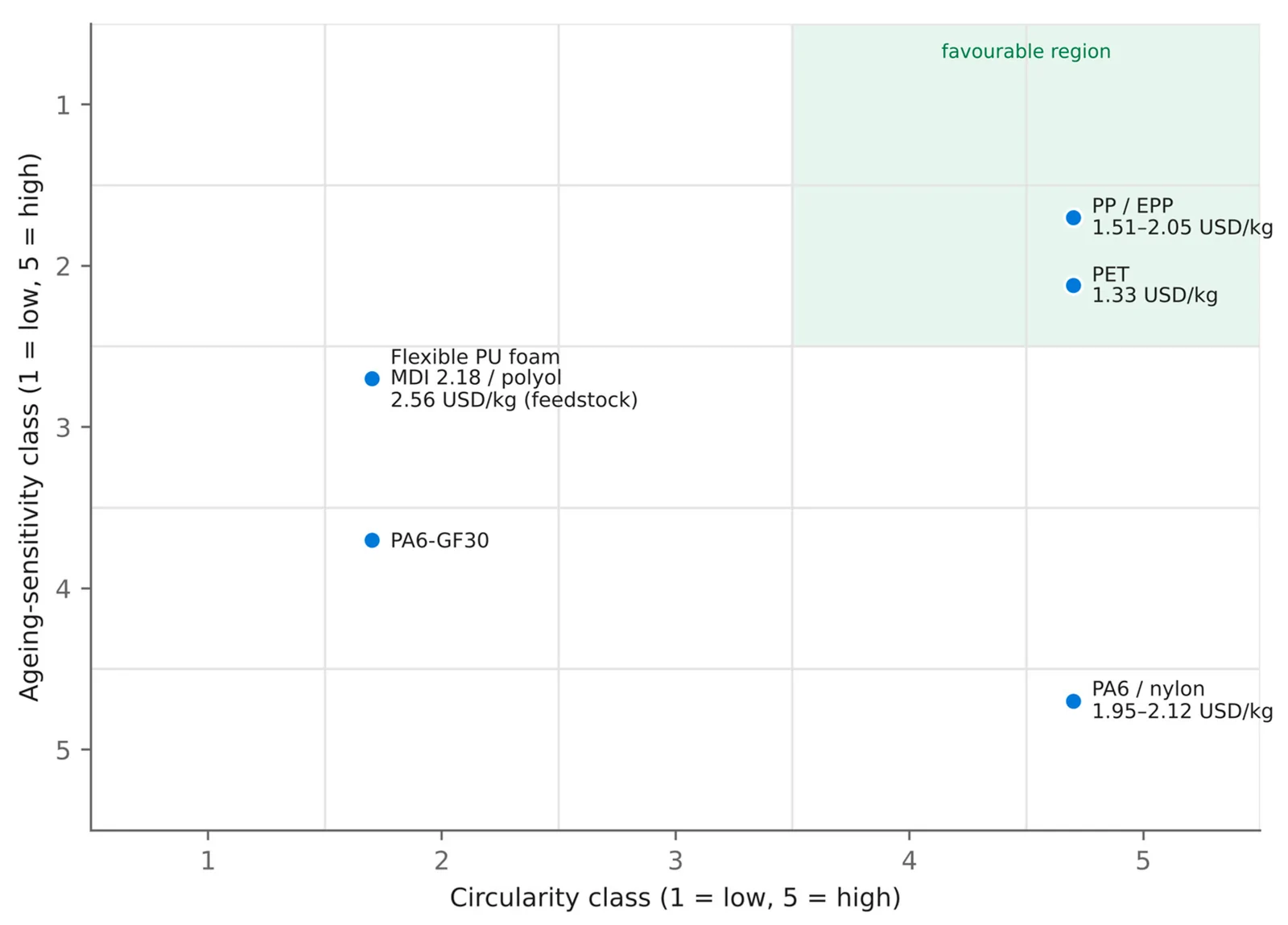
Ageing-sensitivity class versus circularity class of the material families, with indicative virgin-material prices (Table 3 and Table 4). The shaded region marks low ageing sensitivity (class ≤ 2) combined with high circularity (class ≥ 4). For PP/EPP, the higher of the two ageing classes is shown. TPU (ageing class 3; circularity not classified) and PP-GF30 (ageing not classified; circularity class 3) are not placed because one of the two classes is undetermined. Price data were not available for PA6-GF30.

Figure 4 combines the two classes with indicative prices. PP/EPP occupies the favourable region (low ageing sensitivity, high circularity) at 1.51–2.05 USD/kg, together with PET at 1.33 USD/kg; PA6 is equally circular at material level but has the highest ageing sensitivity; and flexible PU foam and PA6-GF30 combine moderate-to-high ageing sensitivity with low circularity. This explains why PP/EPP is preferred for the replaceable structural and energy-absorbing modules in Section 6, but it does not make PP/EPP universally superior: EPP is temperature-sensitive (sub-score A2 = 5), and PP cannot replace high-tenacity textiles in the tensile restraint path.

### 5.4. Recycled Content in Safety-Critical Components

Increasing recycled content must be reconciled with mechanical reproducibility. Automotive PP-glass-fibre composite showed only a slight reduction in elastic modulus and tensile and flexural strength after one recycling cycle [78], and an end-of-life-vehicle-derived recycled PP-GF reached a longitudinal tensile strength of 49.08 MPa and an elastic modulus of 3.84 GPa in material extrusion [80]. By contrast, the impact strength of glass-fibre-reinforced PP decreased from 15.2 to 8.2 kJ/m^2^ (about −46%) when the post-consumer recycled fraction of the matrix reached 70%, which was associated with matrix heterogeneity and titanium-dioxide contamination [58]. Recycled-content percentage alone is therefore a poor predictor of safety performance; the specification should include contaminant control, fibre-length distribution, impact strength, and lower-tail variability. A practical hierarchy is to use high-quality recyclates first in non-critical covers and housings and to introduce them into semi-structural or reinforced parts only after batch variability and aged dynamic performance have been demonstrated; a replaceable EPP or PP module is advantageous because it can be characterised separately.

### 5.5. Design for Disassembly and Material Traceability

A circular canine transport system should be designed around disassembly rather than end-of-life shredding. Recommended measures include polymer identification marks, a digital material passport, mechanical rather than irreversible adhesive joining where feasible, replaceable crash absorbers, detachable washable liners, separable electronics, and avoidance of unnecessary mixed-polymer laminates. Compliance with the EU Regulation on Registration, Evaluation, Authorisation and Restriction of Chemicals (REACH) is useful but not equivalent to full material transparency; suppliers should disclose resin family, relevant substances of very high concern, flame-retardant strategy, coating chemistry, recycled content, and recycling instructions.

## 6. PMSS Framework, Material Architecture, and Validation Programme

### 6.1. Purpose, Non-Compensatory Hard Gates, and Reporting Domains

A materials-centred comparison requires a framework that prevents one strength from concealing a critical weakness. A highly recyclable component should not receive an acceptable overall rating if it loses crash strength after UV ageing, and an exceptionally durable polymer should not receive a high rating if accessible additives exhibit unacceptable migration. The proposed Polymer Material Safety and Sustainability (PMSS) framework therefore uses two layers: non-compensatory hard gates evaluated on the aged material state, and a profile of separately reported evidence domains.

A material system fails, regardless of its performance in other domains, if any of the following occurs within the validated ageing envelope: brittle fracture of a primary structural component; permanent loss of energy-absorber geometry outside the qualified range; rupture of restraint textile; closure or connector failure; chemical migration exceeding an applicable regulatory or product-specific limit; uncontrolled shedding of sharp reinforcing fibres; or inability to separate a replaceable crash-critical component after activation. In the notation of Section 4.5, each gate corresponds either to a property margin falling to zero or to the hard-fail indicator switching to one.

For products that pass all hard gates, the PMSS framework reports six domain outcomes (Table 15) rather than a single weighted total. The purpose is diagnostic: designers and certifiers should be able to see whether a material architecture is limited by mechanical retention, environmental durability, chemical or particulate safety, circularity, hygiene, or traceability. Numerical weighting is deliberately not prescribed, because no empirical dataset currently supports universal weights for these domains.

### 6.2. Positioning of PMSS Relative to Established Methods

PMSS is not intended to replace established material-selection and risk-assessment tools but to combine elements of them for a specific class of ageing, animal-contact safety products. Table 16 compares PMSS with four established approaches: performance-index materials selection using property charts [81], multi-criteria decision-making (MCDM) methods such as the analytic hierarchy process (AHP) and the technique for order of preference by similarity to ideal solution (TOPSIS) [82,83], failure mode and effects analysis (FMEA) [84], and life cycle assessment (LCA) [85,86].

The original contribution of PMSS therefore lies in four features that are absent from, or optional in, the established methods: (i) gates are evaluated on the aged rather than the new material; (ii) time enters explicitly through the normalised property margins and the hard-fail step of Section 4.5; (iii) dog-specific contact exposure is a separate domain rather than an ecotoxicity category; and (iv) the traceability domain lowers the confidence of all other domains when composition is undisclosed. PMSS is conceptual and has not yet been validated experimentally; the worked example below illustrates its use but does not constitute validation.

### 6.3. Worked Example: Selecting the Material of a Replaceable Base Energy Absorber

To illustrate how PMSS guides a technical decision, it is applied to the replaceable energy absorber between the carrier and its ISOFIX base. The design values below are illustrative assumptions (evidence Level IV) chosen to demonstrate the procedure, and the material data are quasi-static values from Table 4; a real design would use dynamic (rate-dependent) crushing data.

Step 1—functional requirement (domain MP). For a combined dog-and-carrier mass of 15 kg and a target mean deceleration of 25 g, the absorber must transmit a mean force of about 3.68 kN; with an available stroke of 40 mm it dissipates 147 J, corresponding to a relative velocity of 4.4 m/s. Because the transmitted force equals the crushing strength multiplied by the loaded area, the required area is 256, 157, and 93 cm^2^ for EPP of 30, 45, and 60 kg/m^3^, respectively (crushing strengths 0.144, 0.235, and 0.397 MPa [28]). With a densification strain of 0.6–0.7 [28], the 40 mm stroke requires a block thickness of about 67 mm. A viscoelastic PU foam with a hardness of 2–3 kPa at 20 °C [33] would need about 1.5 m^2^ of loaded area, which is geometrically impossible; it therefore fails the MP gate as an energy absorber and is restricted to comfort inserts.

Step 2—hard gate on the aged and temperature-conditioned state (domains MP and AD). For EPP of 45 kg/m^3^ sized at 157 cm^2^, the reported collapse-stress changes [31,32] imply a mean deceleration of about 52 g at −30 °C, while at 60 °C only about 74 J (50%) can be dissipated within the 40 mm stroke, so that the remainder would be absorbed after densification. The gate is therefore passed only conditionally: the design must be resized (longer stroke, density grading, or geometric triggering) and verified by sled tests at −30, 23, and +70 °C, before and after the multi-stressor ageing of Section 6.5. A compression set of 3–4% after 24 h recovery [29,30] corresponds to a stroke loss δ ≈ 0.04, i.e., about 4% higher deceleration according to Equation (1), which is acceptable if confirmed after ageing.

Step 3—domain profile. Table 17 reports the PMSS profile of the two candidates. No weighted total is calculated.

Step 4—decision. EPP is selected for the replaceable absorber, with density, area, stroke, and temperature-conditioned dynamic performance as controlled specifications, and the PU foam is restricted to a removable comfort layer. The example shows the added value of PMSS over a performance-index ranking alone: an Ashby index would rank the foams by energy absorbed per unit mass but would not reveal the fourfold temperature variation in the transmitted force, the emission issue, or the replacement logic after crash activation.

### 6.4. Proposed Material Architecture and Its Technical Rationale

The system-level concept of a rigid canine cell attached to an ISOFIX base can be formulated as a materials-design problem whose objective is a controlled load path with minimal material complexity, ageing sensitivity, and end-of-life burden. Table 18 lists the proposed material for each subsystem together with its quantitative rationale, the principal ageing risk, the validation test with its acceptance parameter, and the circular-design measure. The selection follows four rules derived from Section 3, Section 4 and Section 5: (i) materials that carry crash loads must keep their function across −30 to +70 °C and after conditioning (Figure 1, Table 7); (ii) materials in contact with each other should belong to compatible thermoplastic families or be mechanically separable; (iii) sacrificial elements are separate monomaterial modules; and (iv) each subsystem has a test that defines its acceptance limit P_i,min_ for Equation (5).

The rationale for PP/EPP in the structural and energy-absorbing modules is therefore not a general superiority of the material but the combination, documented above, of low moisture uptake, an established recycling loop, a price below those of PA6 and TPE, and compatibility between housing, shell, and absorber within one polymer family. The components that require specific experimental validation before the architecture can be considered proven are the EPP absorber (temperature-conditioned and aged dynamic response), the shell and connectors (low-temperature fracture mode after UV ageing), and the restraint webbing (breaking force after UV, moisture, and abrasion).

### 6.5. Recommended Experimental Validation Programme

**Figure 5:**
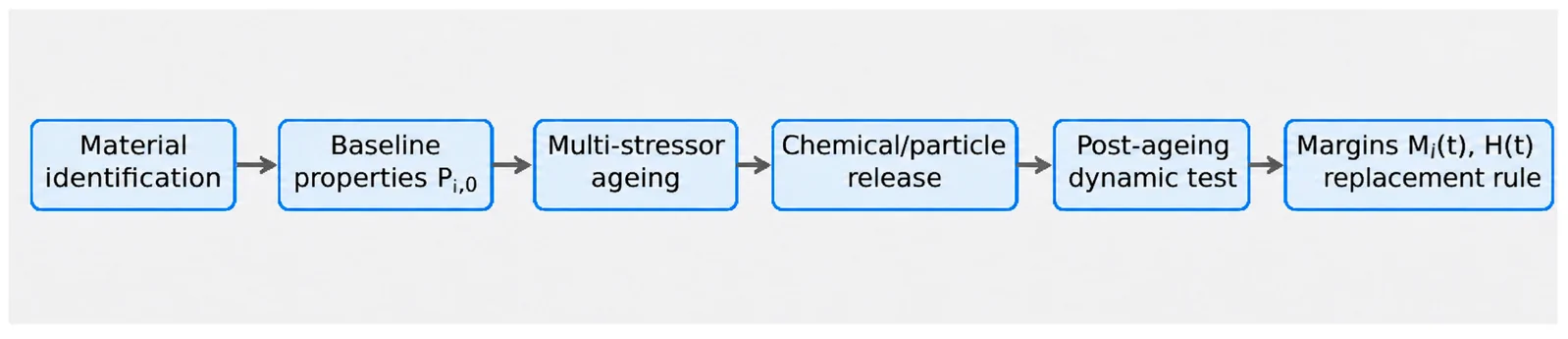
Proposed evidence chain for polymer qualification in canine vehicle restraint systems, linking material identification, baseline properties, realistic ageing, dog-contact exposure assessment, post-ageing dynamic performance, and the final service-life or replacement rule.

The most valuable next step is not another unconditioned crash test of a new product but quantification of how polymer ageing changes crashworthiness and dog-contact exposure over service life. Table 19 proposes a staged programme that can be applied to candidate materials and complete systems, and Figure 5 shows the evidence chain that links the stages to a service-life or replacement rule.

Single-factor ageing identifies mechanisms but does not reproduce the service environment, in which the most severe material state may result from sequential or simultaneous exposure: UV-induced surface oxidation may increase susceptibility to abrasion; moisture may plasticize PA before impact; heat may increase TPU compression set while accelerating VOC emission; and repeated cleaning may remove surface stabilisers. Table 20 therefore defines service scenarios that combine environmental exposure with component function. They are not proposed as finalised certification severities but identify the combinations that should be represented when accelerated protocols are calibrated against field-aged products.

The statistical plan should be defined before testing and should treat material batch, exposure condition, and geometry as controlled factors. For coupon-scale tests, at least five specimens per material–condition combination are recommended as a starting point, because the one-sided 95%/95% tolerance factor used in Equation (5) decreases from 4.20 at *n* = 5 to 2.91 at *n* = 10 and 2.40 at *n* = 20; small groups therefore produce conservative acceptance limits. For destructive sled tests, at least three repeats per configuration give an initial estimate of repeatability. Results should be reported as mean, standard deviation, coefficient of variation, effect size, and confidence interval; multi-factor ageing should be analysed by analysis of variance or regression to separate temperature, UV, moisture, cleaning, and interaction effects; and where the response is monotonic with exposure, degradation curves should be fitted and the uncertainty of the threshold-crossing time propagated into the service-life estimate. For crash-related outputs, a transition from ductile deformation to brittle fracture should be treated as more important than a modest change in a mean scalar metric.

## 7. Discussion, Limitations, and Conclusions

### 7.1. Synthesis of Findings

The central finding of this review is that canine transport systems should be treated as ageing polymer safety systems. Instead of asking only which material is strongest or softest when new, the relevant question is whether the complete material architecture preserves its required load path, energy absorption, surface integrity, and chemical profile after realistic service exposure. The quantitative comparison supports a clear division of roles: EPP for replaceable energy absorbers, impact-modified PP for housings, high-tenacity PET or PA for restraint webbing, TPE/TPU for dampers and feet, flexible PU only for removable comfort inserts, and GFRP only for separable, highly loaded connectors. EPP/PP occupies the most favourable region of the ageing–circularity map at a price (1.51–2.05 USD/kg) comparable to PET and below PA6 and TPE (Figure 4), but this position depends on separable monomaterial design, stabilisation against UV, and specification of density and crush stroke.

The second finding is the material-transparency gap. Product pages may disclose a principal polymer family but rarely provide the resin grade, stabiliser package, flame-retardant chemistry, surface treatment, adhesive system, recycled content, or migration data, although two products both described as “nylon” or “foam” can have very different durability and emission profiles. The third finding is that chemical safety must be treated cautiously: dogs are demonstrably exposed to plastic-associated chemicals and microplastics [60,61,62,63,65], yet there is insufficient evidence to assign that exposure to vehicle products, so source-resolved testing is required. The fourth finding is that service life should be property based, with replacement triggered by a vanishing normalised margin or an irreversible damage event (Section 4.5) rather than by an unsupported calendar interval. The fifth finding is that circularity is decided at the architecture stage: a replaceable EPP absorber, removable PET liner, separable metal anchor, and mechanically fastened thermoplastic shell create a substantially better end-of-life pathway than a bonded laminate.

### 7.2. Limitations

This review has several limitations. First, it is a critical scoping review; pooled effect estimation was not possible because of heterogeneous test methods. Second, the commercial market is large and specifications change, so the selected products are representative examples rather than an exhaustive census. Third, manufacturer declarations rarely provide complete resin grades, stabiliser systems, coatings, adhesives, or additive packages, and no proprietary chemistry was inferred. Fourth, the property values in Table 3 are typical ranges from handbooks, data sheets, and the cited studies; individual grades may fall outside these ranges, and the cost figures are indicative market prices that fluctuate. Fifth, the ageing-sensitivity and circularity classes are ordinal summaries of the indicators in Table 6 and Table 13; they order material families but are not measurements, and different threshold choices could shift a family by one class. Sixth, much of the ageing literature concerns automotive, textile, or general engineering applications rather than canine products; polymer-family mechanisms are transferable, but product-specific lifetime requires direct testing. Seventh, the PMSS framework and the worked example are conceptual and have not yet been validated through interlaboratory experiments or field-aged products. Finally, no dataset was identified that directly correlates accelerated ageing with the post-ageing crash response of canine restraint systems; the links in Section 4.4 are, therefore, partly engineering hypotheses.

### 7.3. Conclusions

Canine vehicle restraint systems are polymer-intensive, multi-material safety products in which energy absorption, structural containment, restraint, cushioning, hygiene, and animal contact depend on composition, morphology, interfaces, and properties that evolve throughout service. EPP/PP, flexible PU foams, PA, PET, TPE/TPU, and GFRP offer complementary functions, but each introduces distinct sensitivities to thermal oxidation, UV radiation, moisture, compression, abrasion, and end-of-life processing.

Service life should be property-based rather than assigned as a universal number of years. A component should remain in service only while every safety-critical property retains a positive normalised margin relative to an acceptance limit derived from matched dynamic tests and while no irreversible damage is present; accelerated-ageing extrapolation is admissible only under the stated Arrhenius validity conditions. Because a reduction in available crush stroke raises the mean deceleration in inverse proportion, and because moisture, low temperature, and recycled-content contamination measurably reduce the impact performance of PA and PP-based materials, ageing must be evaluated through post-ageing dynamic tests rather than inferred from appearance.

Chemical and particulate exposure is a second, currently under-resolved requirement; product-specific synthetic-saliva extraction, heated-chamber VOC/SVOC analysis, and particle-shedding tests after ageing are more defensible than extrapolated toxicity claims. Circularity is determined primarily by product architecture: replaceable monomaterial EPP absorbers, recyclable thermoplastic housings, detachable textile liners, separable hardware, minimal adhesive bonding, and material identification improve repairability and recovery without weakening the safety concept. The decisive research need is a matched dataset of unaged and aged complete restraints tested dynamically after controlled thermal, UV, moisture, cleaning, abrasion, and cyclic-loading histories, which would allow accelerated protocols to be calibrated and defensible service-life specifications to be written.

## Figures and Tables

**Table 1:** Search blocks, search strings as executed in OpenAlex Topics 2.0 on 22 October 2026, searched field, number of records identified, and number of sources included.

Block	Search String (OpenAlex Syntax)	Field	Records Identified	Included
A. Canine transport and restraint	(dog OR canine) AND (crate OR harness OR restraint) AND (vehicle OR car)	Title and abstract	91	9
B. Polymer properties and ageing	(polypropylene OR polyurethane OR polyamide) AND (ageing OR ageing) “expanded polypropylene”	Title	1324 106	12
C. Chemical and particulate exposure	(dog OR canine) AND (phthalate OR bisphenol OR microplastic) (dog OR canine) AND (“flame retardant” OR PFAS) (vehicle OR automotive) AND interior AND (VOC OR volatile)	Title and abstract Title and abstract Title	145 116 44	10
D. Recycling and end-of-life	recycling AND (polypropylene OR polyurethane OR polyamide OR polyester)	Title	4297	17
E. Standards, data sheets, market data, and product declarations	Targeted retrieval from ISO, IEC, and ASTM catalogues, producer data sheets, market-price reports, and manufacturer pages	—	Targeted retrieval (not a database search)	44
Total	—	—	6123 (sum of blocks A–D; overlaps between blocks not removed)	92

PFAS = per- and polyfluoroalkyl substances; VOC = volatile organic compound. Included = number of sources cited in this review that were assigned to each block, including sources obtained by citation chaining.

**Table 2:** Representative canine transport systems: manufacturer-declared material architectures, safety-critical polymer roles, and priority validation needs.

System	Declared Materials	Safety-Critical Polymer Role	Disclosure	Principal Ageing Concern	Priority Validation Need
Tavo Maeve/Maeve iso [7]	EPP structural foam; memory foam; textile canopy; rubberized details; metal/alloy attachments	High: EPP and memory foam linked to energy management	Principal energy-management materials disclosed; additives and coatings not disclosed	Thermal/UV ageing of EPP and textiles; compression set of memory foam; emissions	Replaceable absorber; ageing-conditioned dynamic tests; VOC/SVOC and saliva-migration screening
MIMsafe Care2 [8]	Shatter-resistant nylon; aircraft-grade aluminium; reinforced doors	High: rigid enclosure and crash load path	Polymer family disclosed; grade and additives not disclosed	PA moisture and UV response; hinge/latch interface fatigue	Conditioned PA tests; UV/humidity retention; disassembly study
4pets PRO NOIR [9]	Glass-fibre-reinforced plastic connectors and lock housings; anodized aluminium profiles; polymer tray/walls	High: structural joints and closure	Hybrid architecture and REACH compliance declared; matrix chemistry not disclosed	Composite interface damage; matrix UV/thermal ageing	Joint fatigue after ageing; matrix identification; composite end-of-life route
Sleepypod Clickit Range [10]	Ballistic nylon; neoprene buckle shield; padded webbing vest; metal hardware	High: tensile restraint and load distribution	Textile and elastomer families disclosed; coatings not disclosed	Abrasion; UV; moisture; elastomer ageing	Tensile retention after UV and wet cycling; abrasion; saliva-accessible migration
TRIXIE Car Seat 13176 [11]	Polyester textile; foam padding	Primarily comfort and positioning	Textile family disclosed; foam chemistry not disclosed	Textile shedding; foam compression set	Identify foam polymer; abrasion/shedding; thermal and VOC screening

REACH = Registration, Evaluation, Authorisation and Restriction of Chemicals (EU Regulation No. 1907/2006); VOC/SVOC = volatile/semi-volatile organic compounds.

**Table 3:** Typical properties and indicative prices of solid polymers and fibres used in canine transport systems (23 °C unless stated; d = dry as moulded, c = conditioned at 23 °C and 50% relative humidity (RH)).

Material (Reference Grade)	Density (kg/m^3^)	Tensile Modulus (GPa)	Tensile/Yield Strength (MPa)	Notched Charpy (kJ/m^2^), 23 °C/Cold	Water Absorption (%)	Thermal Data (°C)	Indicative Price
PP impact copolymer (BD310MO) [14,15]	905	1.4	28 (yield)	9/4 (−20 °C)	0.01–0.02 (96 h) ^a^	HDT-B 85; T_g_ ≈ −10 ^a^	1.51–2.05 USD/kg [16]; rPP 0.77–0.78 €/kg [17]
PA6 (B3S HP R01; TECAMID 6) [18,19]	1140	3.2 d/1.0 c	80 d/40 c (yield)	4.9 d, 23 c/3.9 d, 3.4 c (−30 °C)	2.7–3.2 (50% RH); 9–10 (sat.)	T_g_ 45; T_m_ 220; service 100	1.95–2.12 USD/kg [20]; rPA6 1.53–1.55 €/kg [17]
PET (TECADUR PET) [21]	1390	3.3	91	150 (unnotched)	0.02 (24 h); 0.03 (96 h)	T_g_ 81; service 110	1.33 USD/kg [22]; rPET 1.55 €/kg [17]
PP-GF30 (Fibremod GB317SF) [23]	1120	6.3 (flexural)	105	10/9 (−20 °C)	n.d.	HDT-B 160	1.84 USD/kg [24]
PA6-GF30 (Ultramid B3EG6) [25]	1360	9.5 d/6.2 c	185 d/115 c	15 d, 30 c/11 d (−30 °C)	1.9–2.3 (50% RH); 6.3–6.9 (sat.)	HDT-A 210	—
PA6.6 high-tenacity yarn; elongation at break 18–19% [26]	—	—	7.4–8.4 cN/dtex (tenacity)	—	—	—	—
PET high-tenacity yarn; elongation at break 14 ± 1% [27]	—	—	≥8.1 cN/dtex (tenacity)	—	—	shrinkage 5 ± 1% (177 °C)	—

^a^ Values for an unfilled PP grade (TECAPRO MT) [15]. HDT-A/HDT-B = heat deflection temperature at 1.8/0.45 MPa; T_g_ = glass transition temperature; T_m_ = melting temperature; sat. = saturation in water; n.d. = not determined in the sources consulted. USD prices from market-price reports for Europe (December 2025–September 2026); € prices for recycled regranulates from the January 2026 German market report [17].

**Table 4:** Typical properties of cellular and elastomeric materials used for energy absorption, cushioning, and damping.

Material (Reference)	Density (kg/m^3^)	Compressive Response	Compression Set	Temperature Sensitivity	Other Data	Indicative Price
EPP bead foam, 20–60 kg/m^3^ [28]	20/30/45/60	Mean crushing strength 0.115/0.144/0.235/0.397 MPa	—	—	Densification strain 0.6–0.7; SEA 2.256 → 3.481 J/g (20 → 60 kg/m^3^)	n.a.
EPP (ARPLANK 30/45) [29,30]	30/45	80/120 kPa at 25% strain; 140/200 kPa at 50% strain	25% strain, 22 h: 16/14% after 30 min, 3/4% after 24 h recovery	Service temperature 100 °C	Water absorption < 1%	n.a.
EPP, temperature effect [31,32]	—	Collapse stress +110% at −30 °C; −50% at 60 °C (vs. 23 °C)	—	High	—	—
Viscoelastic PU foam [33]	83–100 (commercial 40–80)	Hardness at 40%: 145 kPa (−20 °C), 50 kPa (0 °C), 2–3 kPa (20 °C), 1.5–2.0 kPa (40 °C)	—	T_g_ 10–20 °C; hysteresis > 0.9 (cold) → ≈ 0.6 (40 °C)	—	Feedstocks: MDI 2.18 USD/kg [34]; polyether polyol 2.56 USD/kg [35]
Slow-rebound PU (PORON 4790-92) [36]	192	Compression force deflection at 25%: 2–17 kPa	≤2% (23 °C); ≤10% (70 °C)	—	—	n.a.
TPU ether/ester, 87 Shore A (Elastollan 1185A10/C85A10) [37]	1120/1190	Tensile strength 45/50 MPa; elongation 600/650%	25% (23 °C, 72 h); 45/35% (70 °C, 24 h)	No break in notched Charpy at −30 °C	Water absorption ≈ 2% (ether grade) [38]	2.57–2.63 USD/kg (TPE, proxy) [39]

SEA = specific energy absorption; MDI = methylene diphenyl diisocyanate. The EPP temperature effect is reported in [31,32] for a specific EPP grade and test rate.

**Table 5:** Polymer families, structure–property basis, and candidate functions in canine transport systems.

Material Family	Structure/Property Basis	Quantitative Advantage (Table 3 and Table 4)	Principal Limitation	Candidate Function
EPP	Closed-cell thermoplastic bead foam; density and bead fusion set the crushing plateau	Crushing strength 0.12–0.40 MPa at 20–60 kg/m^3^; water uptake < 1%	Temperature-dependent collapse stress; permanent crush after severe event	Replaceable base absorber; shell impact liner
Impact-modified PP	Semi-crystalline polyolefin with elastomeric phase	905 kg/m^3^; 1.51–2.05 USD/kg; established recycling	Notched impact −56% at −20 °C; oxidative ageing	Base housing; removable shell panels
Flexible PU/memory foam	Crosslinked cellular PU, viscoelastic near T_g_	Hysteresis ≈ 0.8 at 20 °C (damping)	Hardness × 58 between 20 and −20 °C; not remeltable	Removable comfort pad only
PA6/nylon	Hydrogen-bonded engineering thermoplastic and fibre	Yield strength 80 MPa (dry); yarn tenacity 7.4–8.4 cN/dtex (PA6.6)	Strength −50% when conditioned; 9–10% water at saturation	Technical textile; hardware interfaces after conditioned qualification
PET/polyester	Semi-crystalline polyester; T_g_ 81 °C	Water uptake 0.03%; yarn tenacity ≥ 8.1 cN/dtex	UV strength loss in webbing; microfibre shedding	Liners; covers; restraint webbing after validation
TPE/TPU	Phase-separated thermoplastic elastomer	No break at −30 °C; elongation 600–650%	Compression set 35–45% at 70 °C	Feet; seals; replaceable dampers
GFRP (PP or PA6 matrix)	Short glass fibres in thermoplastic matrix	Strength 105 MPa (PP-GF30) to 185 MPa (PA6-GF30, dry)	PA6 matrix moisture-sensitive; fibre shortening in recycling	Connectors; local structural members

**Table 6:** Indicators and thresholds used to derive the ageing-sensitivity class.

Code	Indicator	Metric	Sub-Score 1 (Low)	Sub-Score 3 (Moderate)	Sub-Score 5 (High)
A1	Moisture uptake	Water absorption (ISO 62 or equivalent; saturation or longest reported exposure)	<1%	1–3%	>3%
A2	Low-temperature change of function	Change of notched impact strength (rigid) or compression hardness/collapse stress (foams) between 23 °C and −20/−30 °C	<25%	25–60%	>60%
A3	Permanent set at elevated temperature	Compression set of foams/elastomers after sustained compression (data-sheet method); not applicable (n.a.) to rigid parts	<15%	15–35%	>35%
A4	Documented loss after accelerated ageing	Largest reported loss of a mechanical property after UV, humidity, or heat ageing in the cited studies	<10%	10–30%	>30%

**Table 7:** Indicator values, sources, and resulting ageing-sensitivity sub-scores (→) and classes; (k/4) = number of indicators determined.

Material	A1 Moisture Uptake	A2 Low-Temperature Change	A3 Permanent Set (Hot)	A4 Loss After Ageing	Class
PP (impact copolymer)	0.01–0.02% (96 h) [15] → 1	9 → 4 kJ/m^2^ (−56%, −20 °C) [14] → 3	n.a.	stable tensile; small impact loss (UV, interior rPP) [42] → 1	2 (3/4)
EPP	<1% [29,30] → 1	collapse stress +110% (−30 °C) [31,32] → 5	3–4% (22 h, 25%, 24 h recovery) [29,30] → 1	n.d.	2 (3/4)
Flexible PU (viscoelastic) foam	n.d.	hardness 2–3 → 145 kPa (×58) [33] → 5	≤10% at 70 °C (one slow-rebound grade) [36] → 1	n.d. (oxidation → carbonyl VOCs) [44]	3 (2/4)
PA6/nylon	9–10% (saturation) [18] → 5	23 → 3.4 kJ/m^2^ (−85%, cond., −30 °C) [18] → 5	n.a.	impact −40% (504 h, 50% RH) [45] → 5	5 (3/4)
PET	0.03% (96 h) [21] → 1	n.d.	n.a.	breaking strength ≈ −30% (12 months sunlight, webbing) [55] → 3	2 (2/4)
TPU (87 Shore A)	2% [38] → 3	no break at −30 °C (notched Charpy) [37] → 1	35–45% (70 °C, 24 h) [37] → 5	no change except +2–3 Shore D (10 weeks, 70 °C, dry) [49] → 1	3 (4/4)
PP-GF30	n.d.	10 → 9 kJ/m^2^ (−10%, −20 °C) [23] → 1	n.a.	n.d.	n.c. (1/4)
PA6-GF30	6.3–6.9% (saturation) [25] → 5	15 → 11 kJ/m^2^ (−27%, dry, −30 °C) [25] → 3	n.a.	n.d.	4 (2/4)

n.a. = not applicable (rigid component); n.d. = not determined in the sources consulted; n.c. = not classified (<2 indicators). The PA6 value for A2 refers to the conditioned state, which is representative of service; dry PA6 changes by −20%. The PET A4 value is an industry summary of sunlight-exposure tests on polyester slings [55] (evidence Level III).

**Table 9:** Polymer-focused failure-mode and verification matrix for canine transport systems.

Component/Material	Failure Mode	Likely Cause	Observable Indicator	System Consequence	Verification Method
EPP absorber	Premature densification or permanent crush	High temperature, repeated compression, prior impact, wrong density	Reduced thickness; altered force–displacement plateau	Shorter stroke; higher deceleration	Compression curve; dimensional check; matched dynamic test
PU comfort/impact foam	Compression set, powdering, damping loss	Heat/humidity ageing, repeated loading, cleaners	Thickness loss; cracking; modulus/damping drift	Reduced cushioning; dog-to-shell contact	Compression-set testing according to ISO 1856:2018 [59]; dynamic compression testing; microscopy.
PA webbing or structure	Strength loss, elongation, embrittlement	Moisture, UV, abrasion, notch damage	Fraying; discoloration; mass uptake	Excursion or rupture of restraint path	Conditioned tensile/impact tests; abrasion; UV exposure
PET textile/webbing	Fibre damage and shedding	Washing, abrasion, UV, claws	Fuzzing; mass loss; particle count	Reduced integrity; particle exposure	Martindale abrasion; wash cycles; particle analysis
TPE/TPU damper	Hardening, softening, permanent set	Thermal–mechanical ageing, moisture, hydrolysis	Hardness shift; compression set; hysteresis change	Loss of damping; altered activation threshold	Temperature-conditioned compression/hysteresis test
GFRP connector	Matrix crack, debonding, delamination	Cyclic load, impact, moisture, processing defect	Visible crack; stiffness loss; exposed fibres	Load-path loss; sudden fracture	Microscopy/NDT; proof load; retained-strength test
Adhesive/coating	Debonding, migration, tackiness	Heat, cleaners, UV, incompatible substrate	Peel-strength loss; surface change	Laminate failure; chemical contact	Peel/shear test; FTIR; VOC/migration analysis

NDT = non-destructive testing; FTIR = Fourier-transform infrared spectroscopy.

**Table 10:** Property-based service-life criteria for principal polymer components.

Component/Material	Critical Property (P_i_)	Dominant Ageing/Loading	Hard Replacement Trigger (H = 1)	Recommended Action
EPP/PP energy absorber	Plateau stress; absorbed energy; residual geometry	Thermal/UV ageing; repeated compression; crash	Permanent crush; cracking; force–displacement outside qualified corridor	Replace absorber module
PU/memory foam	Compression set; dynamic modulus; damping	Heat; humidity; oxidation; cleaners	Permanent set; powdering; cracking	Replace removable insert
PA structural part/textile	Tensile/impact strength; elongation	Humidity; wet/dry cycles; UV; abrasion	Fraying; deep cuts; embrittlement	Replace load-bearing component
PET textile/webbing	Tensile strength; seam integrity; shedding rate	UV; washing; abrasion; heat	Cuts; melted fibres; seam damage	Replace textile/webbing
TPE/TPU/elastomer	Hardness; compression set; damping	Temperature cycling; oxidation; hydrolysis	Cracking; tackiness; permanent set	Replace damper/seal
GFRP connector	Stiffness; strength; joint integrity	Moisture; UV; cyclic load; impact	Matrix crack; exposed fibres; loosened joint	Replace connector/subassembly

**Table 11:** Potential chemical and particulate exposure classes relevant to dog-facing polymer components.

Exposure Class	Potential Material Context	Dog-Specific Pathway	Evidence That Dogs Can Be Exposed	Transport-System Evidence Gap
Phthalates/plasticizers	Plasticized polymers, coatings, adhesives, soft components	Licking/mouthing; dust; dermal contact	Leaching from dog toys and training devices [60]; urinary metabolites [61]	No source-resolved data for canine vehicle restraints
BPA and related bisphenols	Certain PC/epoxy systems and legacy consumer materials	Mouthing; dust; contact transfer	Leaching from selected dog products [60]; EDC review [62]	Material identity and migration unknown
Organophosphate flame retardants	Some foam, textile, and flame-retardant formulations	Inhalation; dust; ingestion; dermal	Paired dog food/urine study [63]	Contribution of automotive pet products not quantified
PFAS/repellent chemistry	Some historic or functional textile finishes	Dermal; licking; dust	Discussed in the companion-animal EDC literature [62]	Coating identification needed; non-fluorinated alternatives available [51]
VOC/SVOC	Foams, composites, adhesives, rubbers, coatings	Inhalation in heated cabin/carrier	Temperature-dependent emissions from automotive materials [44,64]	No dog-carrier chamber datasets identified
Microfibres/microplastics	PET/PA textiles; aged polymers; abrasion	Inhalation; grooming/ingestion; mouthing	Textile shedding [46,47]; PET/PC in pet faeces [65]	No transport-product source apportionment

**Table 12:** End-of-life routes and circular-design implications for polymer families used in canine transport systems.

Material	End-of-Life Route	Quantitative Status	Main Barrier	Design-for-Circularity Measure
PP/EPP	Mechanical recycling; reuse of intact modules	rPP regranulate traded at 0.77–0.78 €/kg [17]	Foam collection logistics; mixed additives; contamination	Mark resin; detachable EPP cartridge; avoid overmoulding
PU foam	Mechanical downcycling; glycolysis	14% of EU mattresses recycled (2017) [72]; ≤50% polyol replacement [73]	Crosslinked network; low density; adhesive bonding	Make foam removable; identify chemistry; avoid laminates
PA/nylon	Mechanical recycling; depolymerization to caprolactam [76]	28 t/day depolymerization plant [71]; rPA6 1.53–1.55 €/kg [17]	Coatings; mixed textiles; moisture during reprocessing	Separate textile/hardware; fibre label; avoid incompatible coatings
PET/polyester	Mechanical recycling; glycolysis/methanolysis; textile-to-plastic	Bottle collection 60% [69]; commercial glycolysis [67]; textile requires chain extender [77]	Blends; dyes; coatings; molecular-weight loss	Monomaterial covers/webbing; detachable modules
TPE/TPU	Mechanical reprocessing of compatible grades	No quantitative data identified	Grade mixing; contamination; hydrolysis	Prefer separable thermoplastic elastomer over bonded rubber
GFRP	Grinding; thermal/chemical fibre recovery	Thermal/chemical routes not near commercialization [74]	Fibre shortening; matrix separation; economics	Use only where structurally justified; detachable parts

**Table 13:** Indicators and thresholds used to derive the circularity class.

Code	Indicator	Metric	Sub-Score 1 (Low)	Sub-Score 3 (Moderate)	Sub-Score 5 (High)
C1	Melt reprocessability	Polymer can be remelted and reshaped	Crosslinked (no)	Thermoplastic with reinforcement damaged by reprocessing	Thermoplastic (yes)
C2	Secondary-material market	Published regranulate price or documented industrial recycling stream for the polymer	None documented	Partial stream or niche (<15% of end-of-life arisings recycled)	Traded regranulate with published price
C3	Property retention after mechanical reprocessing	Reported retention of key mechanical properties after one reprocessing cycle	Not reprocessable or <50%	50–90% or only with additives (e.g., chain extender)	≥90% (“slight reduction”)
C4	Chemical/monomer recovery maturity	Most mature chemical route (depolymerization, glycolysis, solvolysis, pyrolysis)	Not commercial	Pilot or limited commercial; non-monomer products	Commercial depolymerization to monomer

**Table 14:** Indicator values, sources, and resulting circularity sub-scores (→) and classes; (k/4) = number of indicators determined.

Material	C1 Melt Reprocessable	C2 Secondary Market	C3 Retention After Reprocessing	C4 Chemical Recovery	Class
PP/EPP	yes → 5	rPP regranulate 0.77–0.78 €/kg [17] → 5	useful properties retained in post-consumer rPP; slight loss after one cycle (PP-GF) [42,78] → 5	pyrolysis (non-monomer) [79] → 3	5 (4/4)
Flexible PU foam	no (crosslinked) → 1	14% of EU mattresses recycled (2017) [72] → 3	not melt-reprocessable → 1	glycolysis: ≈70% polyol yield; ≤50% virgin-polyol replacement [67,73] → 3	2 (4/4)
PA6/nylon	yes → 5	rPA6 regranulate 1.53–1.55 €/kg [17] → 5	n.d.	depolymerization to caprolactam, 28 t/day plant [67,71] → 5	5 (3/4)
PET	yes → 5	rPET regranulate 1.55 €/kg; bottle collection 60% [17,69] → 5	≈2% elongation without, 171% with chain extender [77] → 3	glycolysis/methanolysis at commercial scale (10 kt/yr) [67,70] → 5	5 (4/4)
TPU	yes → 5	n.d.	n.d.	n.d.	n.c. (1/4)
PP-GF30	thermoplastic; fibres shortened [50] → 3	ELV-derived rPP-GF characterised [80] → 3	slight reduction after one cycle [78] → 5	thermal/chemical not near commercialization [74] → 1	3 (4/4)
PA6-GF30	thermoplastic; fibres shortened [50] → 3	n.d.	n.d.	thermal/chemical not near commercialization [74] → 1	2 (2/4)

n.d. = not determined in the sources consulted; n.c. = not classified (<2 indicators).

**Table 15:** PMSS reporting domains, evidence requirements, and decision role.

Code	Domain	Core Evidence	Decision Role
MP	Mechanical and crash-energy performance	Energy absorption; tensile/impact strength; closure integrity; response at −30 and +85 °C	Hard-gate capable; reports retained safety function after ageing (margins M_i_, Equations (3) and (4))
AD	Ageing and durability	Property retention after UV, thermal, humidity, abrasion, cleaning, and cyclic compression; ageing-sensitivity class (Table 6)	Defines the qualified ageing envelope and service-life evidence (Equations (6)–(8))
CS	Chemical and particulate safety	Volatile and semi-volatile organic compound (VOC/SVOC) emission; synthetic-saliva migration; additive disclosure; particle/microfibre release	Hard-gate capable when an exposure limit or material-integrity criterion is exceeded
CR	Circularity and end-of-life	Circularity class (Table 13); separability; recycled content; material identification; replaceable modules	Reports recovery route and disassembly limitations
HU	Hygiene and dog-contact usability	Cleanability; resistance to saliva, urine, and cleaners; odour retention; surface integrity	Reports dog-contact durability and cleaning compatibility
TR	Traceability and transparency	Resin and additive declaration; supplier documentation; ageing envelope; post-crash replacement information	Evidence-quality domain; unresolved composition lowers confidence in all other domains

**Table 16:** Comparison of PMSS with established material-selection and assessment methods.

Method	Question Answered	Treatment of Ageing (Time)	Treatment of Dog-Contact Chemistry	Trade-Off Logic	Element Adopted in PMSS
Ashby property charts and performance indices [81]	Which material maximises a function-specific index (e.g., E^1/2^/ρ, energy per unit mass)?	Not included; properties of new material	Not included	Index ranking for one objective; trade-off surfaces for two	Function-specific indices in domain MP; property ranges (Table 3, Figure 1)
MCDM: AHP, TOPSIS, VIKOR [82,83]	Which alternative is best under several weighted criteria?	Only if a criterion is defined for it	Only if a criterion is defined for it	Compensatory: weak criterion can be offset by strong ones	Not used for safety decisions; optional ranking only after all hard gates are passed and validated weights exist
FMEA [84]	How can a component fail, with which effect and priority?	Implicit through failure causes	Possible as a failure effect	Risk priority from severity, occurrence, detection	Failure-mode matrix (Table 9) and hard-gate logic
LCA [85,86]	What are the life-cycle environmental impacts?	Through service life assumed in the functional unit	Ecotoxicity categories, not animal-contact exposure	Aggregation within impact categories	End-of-life scenario thinking in domain CR
PMSS (this review)	Does the aged multi-material architecture remain safe, low-exposure, and recoverable?	Explicit: margins M_i_(t), hard-fail step H(t), Arrhenius validity rules	Explicit domain CS with synthetic-saliva migration and VOC/SVOC screening	Non-compensatory gates, then a six-domain profile without weighting	—

**Table 17:** PMSS domain profile for the base-absorber example (illustrative design values; material data from Table 4, Table 7 and Table 14).

Domain	EPP, 45 kg/m^3^	Viscoelastic PU Foam	Consequence for Decision
MP (hard gate)	157 cm^2^ × 67 mm meets 25 g at 23 °C; force varies about fourfold between −30 and 60 °C	About 1.5 m^2^ required at 20 °C: fails	PU excluded as absorber
AD	Ageing class 2; A2 = 5 (temperature)	Ageing class 3	EPP requires temperature-conditioned validation
CS	No product-specific VOC/migration data	Autoxidation forms carbonyl VOCs [44]	Emission screening required for both; priority for PU
CR	Circularity class 5; monomaterial cartridge	Circularity class 2; crosslinked	EPP favoured
HU	Water absorption < 1% [29,30]	Requires washable cover	EPP favoured
TR	Density and grade declared on data sheet	Formulation (index) must be declared [44]	Declare density, grade, and additives

**Table 18:** Proposed material architecture for a next-generation ISOFIX-mounted canine safety carrier, with rationale and validation parameters.

Subsystem	Material Strategy	Quantitative Rationale	Principal Ageing Risk	Validation Test and Acceptance Parameter	Circular-Design Measure
Base housing	Impact-modified PP or PP-GF30 in load-bearing ribs	PP: 905 kg/m^3^, 1.51–2.05 USD/kg; PP-GF30 retains 90% of notched impact at −20 °C, 105 MPa strength	Thermo-oxidation; low-temperature toughness of unfilled PP (−56%)	Notched impact at −30 °C after heat and UV ageing; static proof load of ISOFIX interface; no brittle fracture	Monomaterial PP family; mechanical recycling after metal removal
Energy absorber	Replaceable EPP cartridge, 30–60 kg/m^3^	Crushing strength 0.144–0.397 MPa; SEA up to 3.48 J/g; water < 1%	Temperature dependence of collapse stress; permanent crush	Sled tests at −30/23/+70 °C, new and aged; mean deceleration and residual stroke within corridor	Separate monomaterial recycling; replacement after activation (H = 1)
Carrier shell	Ductile PP-based shell with selective GF reinforcement	PP-GF30 vs. PA6-GF30: no moisture-driven strength loss (PA6-GF30: 185 → 115 MPa conditioned)	UV/thermal ageing; notch fracture	Drop/impact at −30 °C after UV ageing; ductile failure mode required	Same polymer family as base; avoid PA/PP mixing
Side-impact insert	EPP or TPU; removable PU memory foam only for comfort	Viscoelastic PU hardness varies ×58 between 20 and −20 °C	Compression set; temperature sensitivity	Dynamic compression at −30/23/+70 °C; compression set after 22 h at 70 °C	Replaceable insert; no bonding
Internal liner	Monomaterial PET textile	Water uptake 0.03%; PET loop with commercial chemical recycling	Microfibre shedding; washing damage	Martindale abrasion and ISO 6330 washing; particle release and tensile retention	Detachable, fibre-labelled
Restraint webbing	High-tenacity PET or PA6.6	Tenacity ≥ 8.1 (PET) and 7.4–8.4 cN/dtex (PA6.6); PET absorbs far less water	UV loss of 6–20% (nylon webbing); abrasion; moisture	Breaking force after UV, wet/dry cycling, and abrasion ≥ P_i,min_ from Equation (5)	Replaceable webbing module; separable hardware
Feet, seals, dampers	TPU (ether type for hydrolysis resistance) where performance permits	No break at −30 °C; compression set 45% (ether) vs. 35% (ester) at 70 °C	Permanent set at high temperature	Compression set and hysteresis at 70 °C, wet and dry	Thermoplastic recovery preferable to crosslinked rubber
Coatings, additives	Low-migration, fully declared, PFAS-free where feasible	No safe level can be assumed without data	Migration; VOC/SVOC; wear-off	Synthetic-saliva migration; TD-GC-MS at 65 °C	Minimise and declare chemistry

**Table 19:** Proposed experimental programme for polymer ageing, exposure, and post-ageing dynamic validation.

Stage	Recommended Method	Primary Output
Material identification	Attenuated total reflectance Fourier-transform infrared spectroscopy (ATR-FTIR); differential scanning calorimetry (DSC); thermogravimetric analysis (TGA); density; microscopy	Polymer family, fillers, thermal transitions, contamination, supplier consistency
Baseline mechanical properties	Tensile/impact tests for rigid polymers; ISO 3386-1 compression stress–strain [87] and ISO 1856 compression set [59] for flexible cellular materials; tear/abrasion for textiles and elastomers	P_i,0_ values and batch scatter for Equations (3)–(5)
UV ageing	ISO 4892-3 fluorescent-UV exposure [88] and/or a validated xenon-arc programme; matched unaged controls	Photo-oxidation, colour change, cracking, strength/toughness retention
Moisture conditioning	ISO 62 water absorption [89]; controlled humidity and wet/dry cycling	Moisture uptake and conditioned mechanical response
Elastomer heat ageing	ISO 188 accelerated heat ageing [90]	Hardness, tensile/tear retention, permanent set
Thermo-oxidative ageing	At least three temperatures with periodic FTIR, oxidation induction time (OIT), DSC, and mechanical tests	Activation energy and Arrhenius linearity check (Section 4.6)
Cleaning and saliva compatibility	Repeated cleaning; ISO 6330 washing/drying for removable textiles [91]; synthetic canine-saliva extraction adapted from [60]	Swelling, cracking, mass change, chemical migration
VOC/SVOC emission	TD-GC-MS and/or HS-GC-MS at 23 °C and at cabin-relevant 65 °C	Temperature-dependent emissions from foams, coatings, adhesives, textiles
Particle/microfibre release	ISO 12947-2 Martindale abrasion [92] plus particle capture, microscopy, and spectroscopic identification	Polymer shedding and the effect of ageing
Dynamic safety after ageing	Sled tests of new vs. aged matched systems; carrier acceleration, excursion, connector and webbing loads	Ageing-induced change in dynamic response and failure mode
End-of-life disassembly	Timed disassembly; mass balance by polymer/metal stream	Separability and replaceability

**Table 20:** Proposed multi-stressor service scenarios for material qualification.

Scenario	Combined Stressors	Most Sensitive Material Classes	Primary Measurable Outputs	Safety Interpretation
Summer parked vehicle	Elevated temperature + solar UV + low air exchange	PP/EPP, PU, TPE/TPU, textiles, coatings/adhesives	Oxidation markers, modulus, compression set, VOC/SVOC emission	Heat/UV-driven embrittlement, damping drift, emission increase
Wet-dog transport	Moisture + saliva salts and proteins + cyclic compression + abrasion	PA/nylon, textiles, foams, metal–polymer joints	Mass uptake, tensile/impact retention, friction, interface condition	Property shift under realistic animal-contact moisture
Cleaning/disinfection cycle	Detergent or alcohol + drying + rubbing/washing	PET/PA textiles, PU liners, coatings	Mass change, tensile retention, surface chemistry, microfibre release	Whether hygiene maintenance degrades safety-critical surfaces
Winter cold start	Low temperature + prior moisture conditioning + immediate dynamic load	Impact-modified PP, TPE/TPU, PA, GFRP matrix	Impact strength, fracture mode, damping	Low-temperature loss of toughness or compliance
Long-term seating/compression	Static pre-load + cyclic loading + elevated temperature	PU foam, EPP inserts, TPE/TPU dampers	Compression set, plateau stress, geometry recovery	Reduction in available crush stroke (Section 4.4)
Post-crash condition	High-rate loading + permanent deformation	EPP, connectors, webbing, GFRP, latches	Residual geometry, crack inspection, retained strength	Replace-versus-reuse rule after a severe event

## Data Availability

No new data were created or analyzed in this study. Data sharing is not applicable to this article.

## Abbreviations

The following abbreviations are used in this manuscript

AD	Ageing and durability (PMSS domain)
AF	Acceleration factor
AHP	Analytic hierarchy process
ATR-FTIR	Attenuated total reflectance Fourier-transform infrared spectroscopy
BPA	Bisphenol A
CR	Circularity and end-of-life (PMSS domain)
CS	Chemical and particulate safety (PMSS domain)
DSC	Differential scanning calorimetry
EDC	Endocrine-disrupting chemical
EPP	Expanded polypropylene
FMEA	Failure mode and effects analysis
GFRP	Glass-fibre-reinforced polymer
HDT	Heat deflection temperature
HS-GC-MS	Headspace gas chromatography–mass spectrometry
HU	Hygiene and dog-contact usability (PMSS domain)
IEC	International Electrotechnical Commission
ISO	International Organization for Standardization
ISOFIX	International Organization for Standardization FIX child-restraint anchorage system
LCA	Life cycle assessment
MCDM	Multi-criteria decision-making
MDI	Methylene diphenyl diisocyanate
MP	Mechanical and crash-energy performance (PMSS domain)
NDT	Non-destructive testing
OIT	Oxidation induction time
PA	Polyamide
PA6-GF30	Polyamide 6 with 30 wt% short glass fibres
PC	Polycarbonate
PET	Poly(ethylene terephthalate)
PFAS	Per- and polyfluoroalkyl substances
PMSS	Polymer Material Safety and Sustainability
PP	Polypropylene
PP-GF30	Polypropylene with 30 wt% short glass fibres
PRISMA-ScR	Preferred Reporting Items for Systematic Reviews and Meta-Analyses extension for Scoping Reviews
PU	Polyurethane
REACH	Registration, Evaluation, Authorisation and Restriction of Chemicals
RH	Relative humidity
rPP/rPA6/rPET	Recycled PP/PA6/PET
SEA	Specific energy absorption
SVOC	Semi-volatile organic compound
TD-GC-MS	Thermal-desorption gas chromatography–mass spectrometry
TGA	Thermogravimetric analysis
TOPSIS	Technique for order of preference by similarity to ideal solution
TPE	Thermoplastic elastomer
TPU	Thermoplastic polyurethane
TR	Traceability and transparency (PMSS domain)
TRL	Technology readiness level
UV	Ultraviolet
VOC	Volatile organic compound
